# A Nonbenzenoid Nanographene Horse Saddle With Four Pentagon–Octagon Pairs

**DOI:** 10.1002/anie.2412282

**Published:** 2026-06-04

**Authors:** Qiang Huang, Yubin Fu, Zongbao Zhang, Jinjin Ding, Mengyuan Yu, Xinyang Ge, Boris Borrisov, Hartmut Komber, Zhen‐Lin Qiu, Yana Vaynzof, Ji Ma, Xinliang Feng

**Affiliations:** ^1^ School of Chemistry and Chemical Engineering Nantong University Nantong China; ^2^ Max Planck Institute of Microstructure Physics Halle (Saale) Germany; ^3^ Leibniz‐Institute for Solid State and Materials Research Dresden Dresden Germany; ^4^ Chair for Emerging Electronic Technologies Technical University of Dresden Dresden Germany; ^5^ Faculty of Chemistry & Food Chemistry, Technical University of Dresden Dresden Germany; ^6^ Leibniz‐Institut For Polymerforschung Dresden eV. Dresden Germany; ^7^ Beijing National Laboratory for Molecular Sciences CAS Key Laboratory of Organic Solids Institute of Chemistry Chinese Academy of Sciences Beijing China

**Keywords:** negatively curved nanographene, non‐hexagonal rings, optoelectronic devices, precision synthesis

## Abstract

The incorporation of non‐hexagonal rings into nanographenes (NGs) would significantly alter their geometry and electronic structures, unlocking new application potential. However, the precise integration of consecutive pentagon–octagon into NGs remains synthetically challenging due to the associated high strain and the limitation in design strategy. Herein, we report an unprecedented horse‐saddle non‐benzenoid NG (**HN1**) embedding four pairs of pentagon–octagon units. The synthesis proceeds through two key steps: a tetramerization of 3‐bromoacenaphthylen‐1(2*H*)‐one, followed by a three‐fold Pd‐catalyzed annulation with a diacetylene derivative. Single‐crystal X‐ray diffraction confirms that **HN1** adopts a unique horse‐saddle geometry induced by the four adjacent octagons. The fused pentagon–octagon pairs of NG impart configurational stability, high solubility, multiple reversible redox behavior, and a distinct antiaromatic character arising from the core structure. Notably, the incorporation of **HN1** as an additive in the perovskite active layer significantly enhanced the power conversion efficiency (PCE) of perovskite solar cells from 20.60% to 22.61%. This work not only provides a synthetic approach to negatively curved NGs with contiguous nonhexagonal motifs but also explores their promising application in emerging optoelectronic devices.

## Graphical abstract

Horse‐saddle molecule: We report herein the design and synthesis of an unprecedented saddle‐shaped non‐benzenoid Nanographene featuring a fused four‐pair pentagon–octagon core. The horse‐saddle structure of **HN1** is determined by single‐crystal X‐ray diffraction. Investigations into perovskite solar cells have revealed the potential of these materials for applications in optoelectronic devices.

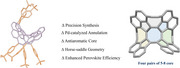

## 1 Introduction

Bottom‐up synthesis enables the construction of new types of precision graphene nanostructures, or nanographenes (NGs), with tailored geometries and properties beyond those of all hexagonal systems [1, 2, 3, 4, 5, 6]. For instance, the introduction of non‐hexagonal rings dictates molecular curvature: five‐membered rings induce positive curvature [7, 8], as seen in fullerenes, whereas heptagons [9, 10, 11], octagons [12, 13, 14, 15, 16, 17], and larger rings impart negative curvature, leading to saddle‐shaped or porous architectures. The nonplanar structure of these negatively curved NGs effectively inhibits π–π stacking, which not only endows them with remarkable solubility but also enhances solid‐state luminescence [4, 18], alongside tunable electronic energy levels and unique magnetic properties. These features make such NGs highly promising for applications in opto‐electronics and quantum materials [19, 20, 21].

Among various non‐benzenoid motifs, NGs that incorporate fused pentagon–octagon pairs represent a particularly intriguing platform for constructing stable negative curvatures, [22]. These structures not only confer pronounced saddle‐shaped geometries but also lead to distinctive electronic properties, including tunable optical gaps and emergent open‐shell character, which are highly desirable for advanced carbon‐based electronics. Despite these compelling attributes, the synthesis of NGs containing multiple pentagon–octagon pairs remains significantly underdeveloped relative to other non‐benzenoid systems. This lag is largely due to the considerable accumulated strain and unresolved regioselectivity challenges involved in forming successive non‐hexagonal rings [14, 23, 24].

**FIGURE 1:**
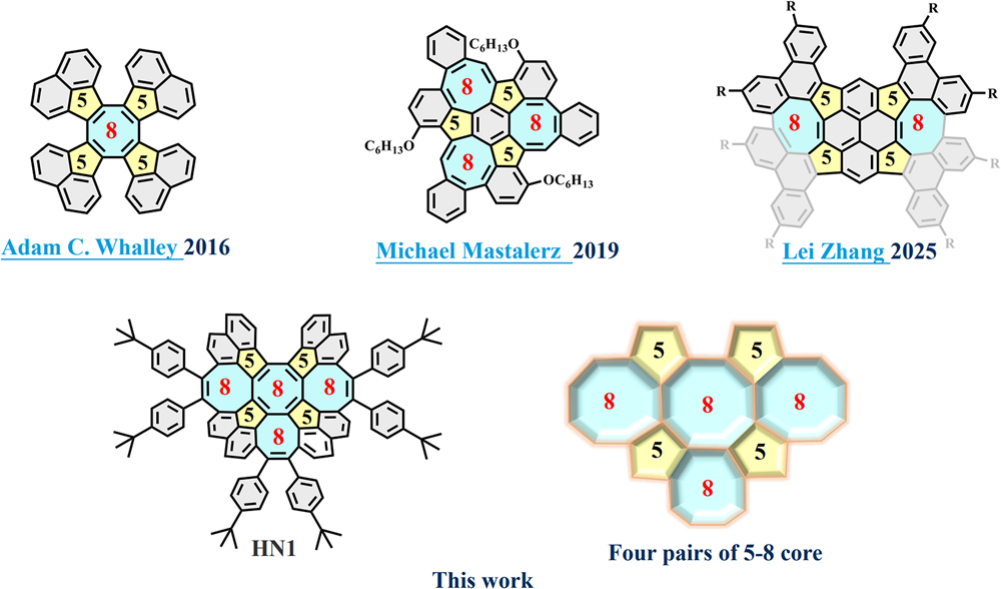
Recent examples of pentagon–octagon embedded NGs and **HN1** with four pairs of pentagon–octagon units in this work.

Amidst these overarching synthetic challenges, the precise and controlled formation of the eight‐membered ring is often the most formidable hurdle. To date, several methods for incorporating octagons into NGs have been established, such as cyclotetramerization [23], dual C–H activation [25], and multi‐step coupling‐condensation strategies [26], among others. As depicted in Figure 1, in 2016, Whalley and colleagues developed an annulative coupling via C–H functionalization to form an octagon from polyaromatic precursors [23]. In 2019, Mastalerz and coworkers employed a multi‐step coupling‐condensation strategy to construct a chiral monkey‐saddle‐shaped NG containing three eight‐membered rings via Suzuki coupling of tribromoindene followed by base‐catalyzed condensation [27]. Very recently, Zhang et al. reported the synthesis of a π‐extended pyrene‐based antiaromatic buckybowl featuring fused pentagon–octagon rings, which exhibit notable host‐guest interactions [28]. Despite these advancements, the synthesis of non‐benzenoid NGs containing a high density of pentagon–octagon pairs remains largely elusive, which limits further exploration of their unique properties for device applications.

**FIGURE 2:**
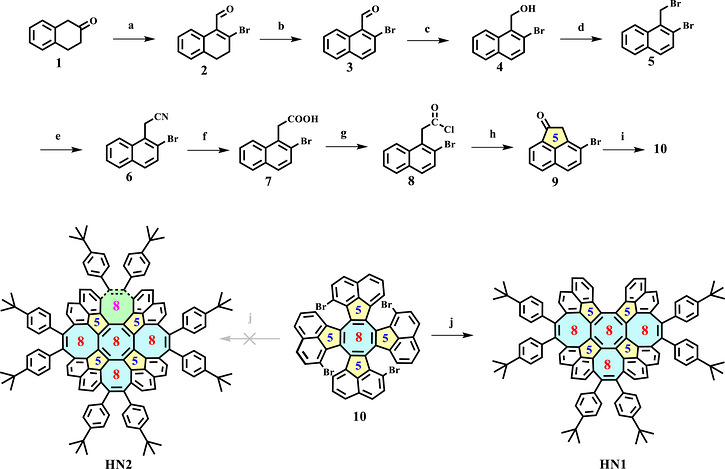
Synthetic route toward **HN1**. Reagents and conditions: (a) PBr_3_, CHCl_3_, DMF, 0°C, 1 h, 85%; (b) DDQ, Toluene, 100°C, 48 h, 80%; (c) NaBH_4_, EtOH, 0°C, 1 h, 93%; (d) PBr_3_, CHCl_3_, 55°C, 8 h, 80%; (e) KCN, DMF, 8 h, 80°C, 66%; (f) KOH, EtOH, H_2_O, 80°C, 24 h, 72%; (g) SOCl_2_, 85°C, 1 h; (h) AlCl_3_, 0°C, 69% over two steps (g, h); (i) TiCl_4_, o‐dichlorobenzene, 180°C, 2 h, 18%. (j) 1,2‐bis(4‐(tert‐butyl)phenyl)ethyne, Pd(OAc)_2_, tri‐(4‐Clorophenyl)‐phosphine, AgOAc, 80°C, 36 h, 32%.

Herein, we demonstrate the synthesis of a previously unreported horse‐saddle non‐benzenoid nanographene (**HN1**), achieved through a strategic fusion of four pentagon–octagon pairs to induce a stable negative curvature (Figure 2). To the best of our knowledge, this structure exhibits the highest density of five‐ and eight‐membered ring frameworks reported to date; specifically, it contains the maximum number of fused five‐membered and eight‐membered rings within a single molecule (Figure S1). The chemical structure of **HN1** was comprehensively confirmed using NMR spectroscopy, HR‐MS, and single‐crystal X‐ray diffraction. Its optical properties were characterized through UV/Vis spectroscopy and supported by DFT calculations. Single‐crystal X‐ray diffraction revealed significant bond‐length alternation within the octagons (1.34–1.51 Å), contrasting with the homogeneous bond lengths of the outer benzenoid hexagons (average 1.40 Å). **HN1** was then explored as a functional additive in the active layer of perovskite solar cells. Its inclusion not only significantly boosted the power conversion efficiency from 20.60% to 22.61%, suggesting optimization of charge transport within the active layer, but also notably improved device‐to‐device reproducibility, underscoring the promise of strategically designed, negatively curved NGs for potential applications in optoelectronic devices.

Our synthetic strategy centers on the key precursor, tetrabrominated cyclooctatetraacenaphthylene core (**10**), which serves as a pivotal intermediate. First, commercially available 3,4‐dihydronaphthalen‐2(1*H*)‐one (**1**) was reacted with PBr_3_ in DMF in CHCl_3,_ affording compound **2**. Next, compound **2** was aromatized with DDQ in toluene at 100^°^C, affording 2‐bromo‐1‐naphthaldehyde (**3**) in good yield. Subsequently, reduction of aldehyde **3** afforded alcohol **4** as a white solid, which, upon reaction with PBr_3_/CHCl_3_, produced bromide **5** as a white solid in 80% yield. The bromide was then converted to the nitrile derivative **6**. Upon hydrolysis, compound **7** was obtained in good overall yield. Compound **7** was treated with thionyl chloride to convert its carboxylic acid group into the corresponding acyl chloride, yielding compound **8**. This intermediate then underwent Friedel‐Crafts acylation mediated by aluminum chloride, effectively constructing a pentacyclic system in compound **9**. Subsequent cyclization of compound **9** in 1,2‐dichlorobenzene solvent, facilitated by titanium tetrachloride, generated the eight‐membered ring architecture of pivotal intermediate **10** in an 18% yield. Compound **10** was first confirmed through MALDI‐TOF mass spectrometry, which shows a main peak at m/z 915.8255 corresponding to the expected molecular weight (Figure S2). The ^1^H and ^13^C NMR spectra of **10** are very complex and characterized by a large number of overlapping signals (Figures S7 and S8). The 2D HSQC and HSQC‐TOCSY spectra reveal five distinct sets of protons corresponding to the five groups of hydrogen in the compound **10** (Figures S9 and S10). This is due to the occurrence of different stereoisomers resulting from different arrangements (up or down) of the five‐membered rings on the central eight‐membered ring. The relevant bonds are highlighted in color in the formula (Figure S7). Variable temperature NMR reveals the structural stability of **10** (Figure S11). Finally, a palladium‐catalyzed reaction between compound **10** and 1,2‐bis(4‐(*tert*‐butyl)phenyl)ethyne was performed using Pd(OAc)_2_, as the catalyst, potassium acetate as the base, and tri‐(4‐chlorophenyl)‐phosphine as the ligand. Although the target molecule **HN2** was not observed despite extensive optimization of reaction conditions, compound **HN1** with the three‐fold annulation was successfully obtained in 32% yield. We attribute this to the significant structural strain induced by the formation of **HN1**, coupled with an increased distance between the reactive sites required for the fourth annulation to construct an additional eight‐membered ring (Figure S12 and S13). This unfavorable geometry likely promotes competing debromination of the intermediate.

Compound **HN1** was first confirmed through MALDI‐TOF mass spectrometry (Figure S3). The main peak at m/z 1465.7542 for **HN1** was observed and matched the calculated value (1465.7546). All the observed signals in ^1^H NMR Spectrum for compound **HN1** can be well assigned with the help of 2D NMR spectra (Figures S14–S20). The ^1^H NMR of **HN1** exhibits a down‐field resonance at *δ* = 8.45 ppm, which corresponds to the proton in the bay region of the molecule. The significant downfield shift is a result of the molecule being saddle‐shaped in geometry.

**FIGURE 3:**
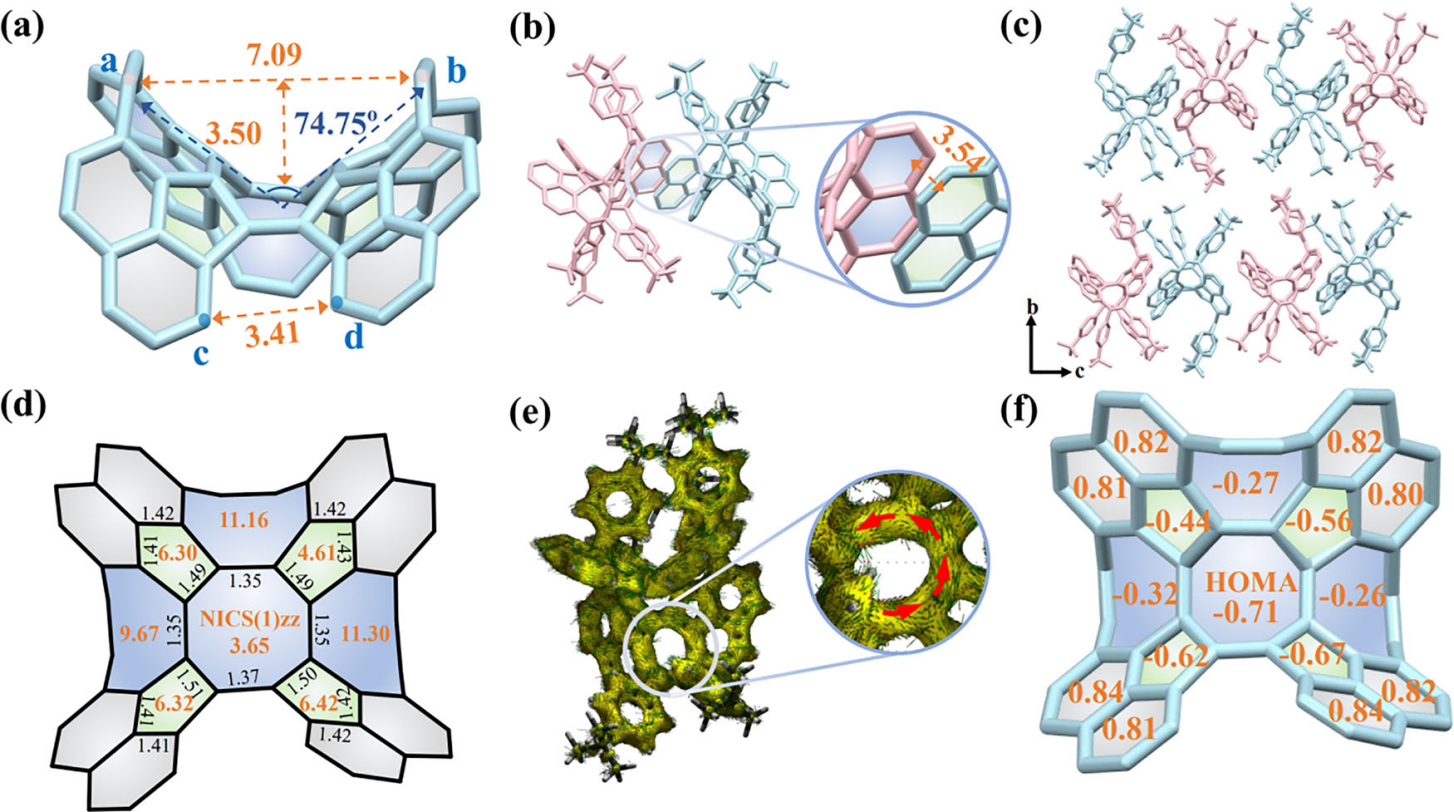
(a) Single‐crystal X‐ray diffraction analysis of **HN1**, depicting the dimensions of the saddle. Molecular structure with thermal ellipsoids at 50% probability. Bond lengths (Å) observed in the X‐ray structure of **HN1**. (b) Visualization of the intermolecular interactions between the two naphthalene planes. (c) Packing structure. All hydrogen atoms are removed for clarity. Solvent molecules are omitted for clarity. (d) Observed bond lengths (mean values, Å) and NICS(1)_ZZ_ values of the non‐benzenoid rings of **HN1**. 1; 4‐tert‐butylphenyl groups are omitted for clarity. (e) Calculated ACID plots of **HN1**. The direction of the magnetic field is orthogonal to the XY plane and points upward. (f) HOMA values of the rings in the molecule of **HN1**.

Single crystals of **HN1** were obtained by slow vapor diffusion of methanol in a chlorobenzene solution of **HN1**. The molecule with the unprecedented horse‐saddle architecture was unambiguously confirmed by single‐crystal X‐ray diffraction. **HN1** crystallized in the space group P 21/n with eight molecules in the unit cell. The detailed crystallographic data are summarized in Table S1. As shown in Figure 3a, the saddle height of the skeleton of **HN1** is measured to be 3.50 Å, and the skeleton width is 7.09 Å (*a* → *b*). As shown in Figure 3a and Figure S21, the direct distance of the molecular bay area increases from 3.02, 3.04, and 3.05 Å to 3.41 Å (*c* → *d*), which may be a key reason for the difficulty in forming the additional octagon ring. In addition, the bending angle between consecutive octagons of the central octagon ring is 74.75°. Furthermore, **HN1** was studied theoretically by means of DFT calculations at the B3LYP‐D3(BJ)/6‐31G(d) level. The geometry optimization of **HN1** (Figure S23) proved its horse‐saddle configuration, in agreement with the crystal structure (Figure S21). The intermolecular distance is 3.54 Å due to the edge‐to‐face π stacking (Figure 3b). The solid‐state packing of the molecule **HN1** is determined by the intermolecular π···π interactions, resulting in a continuous 1‐dimensional structure (Figure 3c). As shown in Figure 3d and Figure S22, the fused part of pentagons and octagons is increased to 1.34–1.51Å due to the geometrical constraints of the ideal bond angle of the pentagons and the distorted conformation of octagons. In contrast, a distinct bond‐length alternation is observed within the octagons, indicative of the strain induced by their nonbenzenoid geometry and the resulting localization of π‐electrons. The six bonds shared by pentagons and hexagons are approximately consistent, ranging from 1.41 to 1.43Å, demonstrating a delocalized double‐bond character. The outer eight six‐membered rings have nearly homogeneous bond‐length distributions (average 1.40 Å) with nonsignificant bond‐length alternation, which indicates the benzenoid character. The nucleus‐independent chemical shift (NICS) (Figure 3d), anisotropy of the current‐induced density (ACID) (Figure 3e), and harmonic oscillator model of the aromaticity (HOMA) (Figure 3f) calculations were performed to evaluate the aromatic character of the horse‐saddle molecule. NICS(1)_ZZ_ presents the negative value of the shielding tensor component perpendicular to the direction of the ring plane, measured at 1 Å above or below the ring plane. Considering the nonplanarity of the two species, the most representative planes of the rings in **HN1** were determined using the least‐square method with the assistance of Multiwfn software. These planes were further utilized for NICS calculations. All the five‐ and eight‐membered rings in **HN1** have fairly positive NICS(1)_ZZ_ values (Figure 3d), indicative of their strong antiaromatic nature. The diamagnetic ring currents in the five‐ and eight‐membered rings were identified from the ACID plots (Figure 3e); the strong counterclockwise (paramagnetic) current flows are indicated by the red arrows. The corresponding harmonic oscillator model of the aromaticity (HOMA) values of outer hexagons is 0.82 on average. Four pentagons and four octagons exhibit significantly higher degrees of antiaromaticity, with negative HOMA values ranging from −0.26 to −0.71. (Figure 3f). In the core of the horse‐saddle molecule, the four fused octagons exhibit pronounced bond length alternation. Combined with positive NICS values, low multicenter bond indices, and the paratropic ring current visualized by ACID, this bond alternation reflects the system's attempt to escape from antiaromatic destabilization, rather than indicating a simple olefinic character.

**FIGURE 4:**
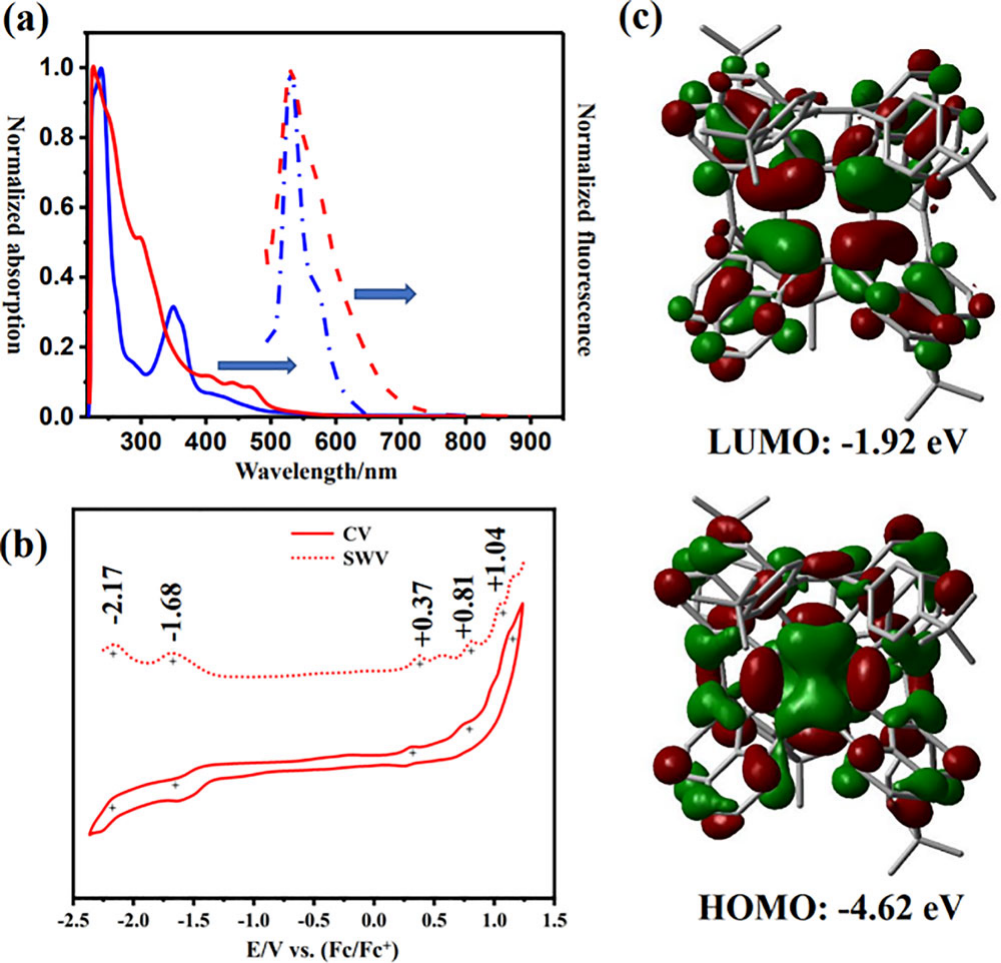
(a) Normalized UV‐vis absorption (solid) and fluorescence spectra (dash dot) of **10** (blue) and **HN1** (red) in CH_2_Cl_2_ solution. (b) Cyclic voltammogram (CV, solid) and square‐wave voltammetry (SWV, dotted) measurements of **HN1** with *c*∼10^−3^ M and a scan rate 50 mV s^−1^. (c) Calculated electron density distributions of HOMO and LUMO for **HN1**.

The photophysical properties of compounds **10** and **HN1** were characterized in CH_2_Cl_2_ solution (1 × 10^−5 ^M; Figure 4a). The absorption bands of **10** were observed between 260 nm and 500 nm, and the maximum absorption peak (l_max_) was observed at 356 nm. Interestingly, the absorption spectrum of **HN1** has a broad shoulder in the visible region between 400 and 500 nm. The selected TD‐DFT‐calculated data for **HN1** are presented in Figure S25 and Table S4. According to the TD‐DFT calculated results, the observed absorption peak of **HN1** at 490 nm and 505 nm (corresponding to the experimental absorption peak at 400–500 nm of **HN1**) is mainly owed to the electron transition of the frontier orbitals of HOMO → LUMO+3 (contribution > 93%), HOMO‐1 → LUMO (contribution > 95%), HOMO‐2 → LUMO (contribution > 90%), and HOMO‐3 → LUMO (Table S4). Compared with the absorption of **10**, this absorption feature in the visible region could be ascribed to the formation of another three octagonal rings. The fluorescence spectra of **10** exhibited a *λ*_max_ at 532 nm. For **HN1**, the emission feature is similar to that of **10** but slightly redshifted, probably due to its extended π‐conjugation. The fluorescence quantum yield (QY) of **HN1** (43.52%) is substantially higher than that of **10** (9.42%) (Figure S26). The cyclic voltammetry (CV) experiment (Figure 4b) was performed in degassed dry CH_2_Cl_2_. The spectra were recorded with a scan rate of 50 mV s^−1^. The square wave voltammetry (SWV) experiments were obtained by using the same solutions. **HN1** shows three oxidations at +0.37, +0.81, and +1.04 V, and two reductions at −1.68 and −2.17 V. This multivalence could positively influence the perovskite active layer by enabling more efficient charge transfer and improved interfacial stability and boosting device performance. We calculated the HOMO and the LUMO energies of **HN1** to be −4.62 and −1.92 eV, respectively (Figure 4c). The HOMO‐LUMO gap of 2.70 eV for **HN1** is consistent with the experimentally observed UV/Vis absorption spectrum (Figure 4a).

**FIGURE 5:**
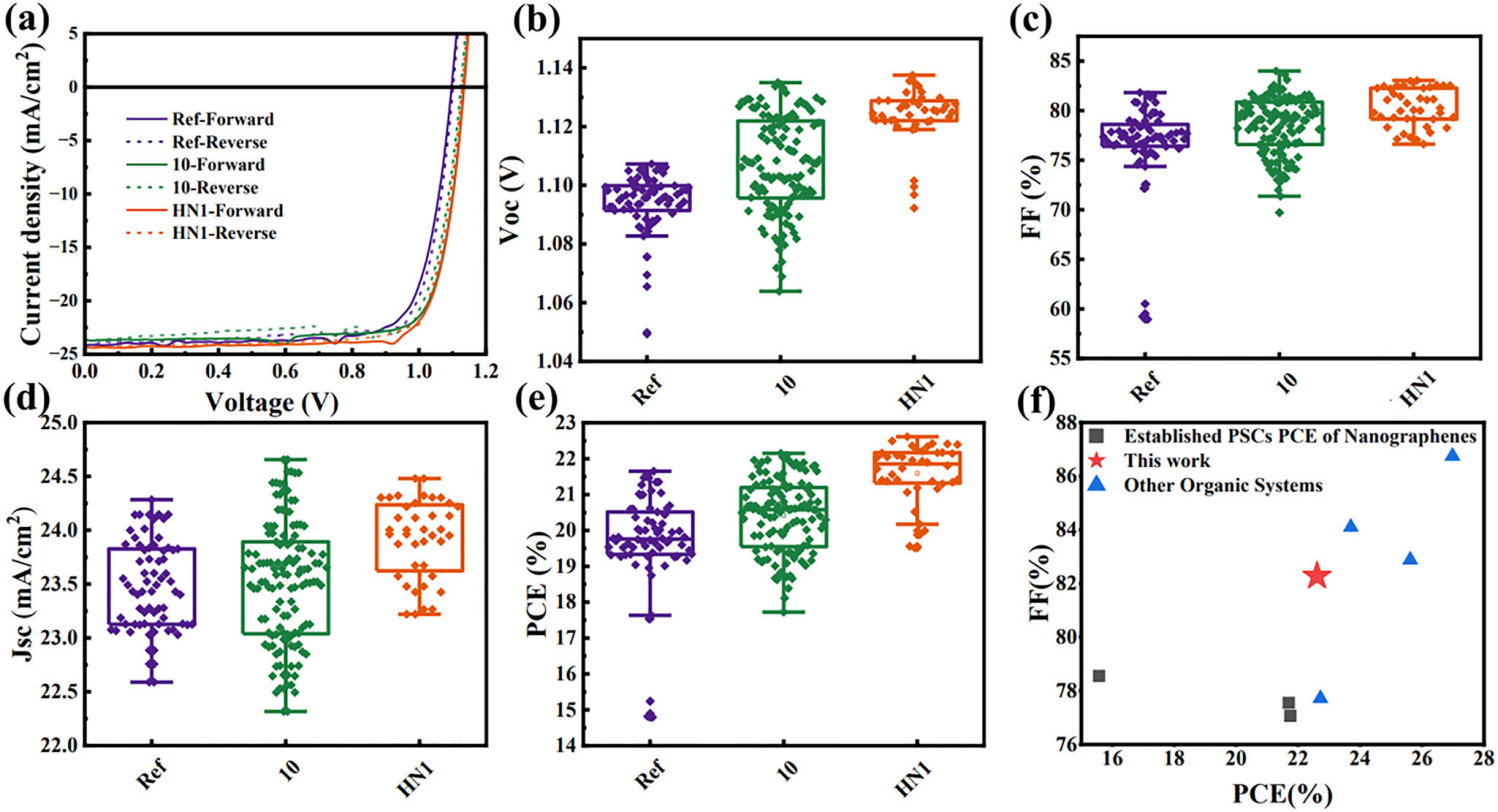
(a) *J‐V* curves of champion perovskite devices of different kinds of additives. Photovoltaic performance parameters: (b) V_OC_, (c) FF, (d) J_SC_, and (e) PCE distributions of perovskite devices with different additives. (f) Photovoltaic performance parameters of champion perovskite devices with different additives. A total of 238 devices were measured.

The nonplanar structure suppresses π–π stacking, enabling remarkable solubility and superior film‐forming ability, offering new possibilities for carbon‐based optoelectronics [6, 8, 29]. To investigate the role of synthesized materials **10** and **HN1** in optoelectronic devices, we incorporated **10** and **HN1** into the perovskite precursors to make perovskite solar cells. The devices are based on a *p*–*i*–*n* structure with an ITO/NiO_x_/Me‐4PACz/perovskite/PCBM/BCP/Ag architecture. The current‐density‐voltage (*J–V*) curves of the champion devices and the distribution of the photovoltaic parameters are illustrated in Figure 5a–e. Figures S29 and S30 show the optimized concentrations of **10** and **HN1** in perovskite precursors. For both additives, an optimized loading amount of 0.25 mg affords the best device performance. Compared with the reference devices, the modified perovskite solar cells exhibit simultaneously enhanced open‐circuit voltages (V_OCs_) and fill factors (FFs), resulting in an improved efficiency. In particular, devices with **HN1** additives deliver a maximum PCE of 22.61% (Table S5). This value not only surpasses that of the precursor **10**, suggesting a positive correlation between octagon content and device performance, but also exceeds the previously reported benchmark of 20.59% for nanographene‐based additives in the same device architectures [30]. As shown in Figure 5f and Table S6, **HN1** achieves higher efficiency than existing nanographenes. Using our molecules as additives helps passivate defects and improve stability, which compensates for the differences compared to other systems. Relative to the precursor **10** containing one eight‐ring, **HN1** features an increased number of octagons, which increases the nonplanarity of the nanographene framework. **HN1** enhances the crystallization and morphology of perovskite films, as evidenced by AFM, XRD, and EIS analyses, leading to improved carrier transport efficiency and device performance (Figure S31). The resulting nonplanar structure effectively suppresses π–π stacking, thereby improving the additive's solubility in the precursor solution and enabling the formation of higher‐quality perovskite films. Moreover, this suppresses nonradiative recombination losses and further enhances photovoltaic performance. Furthermore, perovskite devices incorporating these additives exhibit negligible hysteresis (Figure 5a). Figure S32 displays the external quantum efficiency (EQE) spectrum and the integrated short‐circuit (*J*_SC_) curve for champion perovskite devices with or without **HN1** additives. The integrated *J*_SC_ values are in excellent agreement with the value extracted from the J–V curve, confirming the reliability of the photovoltaic measurements. As shown in Figure S33, after 24 days the reference device retained ∼83% of its initial PCE, whereas the device with **HN1** retained > 93%. Overall, these results demonstrate that both the synthesized **10** and **HN1** play a beneficial role in enhancing the performance of the perovskite devices.

In summary, we have presented the successful synthesis of a novel horse‐saddle nanographene **HN1** containing four pairs of pentagon‐octagon cores, constructed by a Pd‐catalysis reaction from the precursor **10**. Theoretical calculations and single‐crystal X‐ray diffraction reveal that **HN1** adopts an unprecedented horse‐saddle conformation. This unique architecture endows the nanographene with remarkable configurational stability and high solubility, a distinct antiaromatic character arising from the core structure, a narrow optical bandgap (consistent with electrochemical HOMO‐LUMO data), and multiple reversible redox characteristics. Collectively, these features indicate promising film‐forming ability, effective interfacial charge regulation, and defect passivation. Leveraging these advantageous opto‐electronic properties, **HN1** was successfully incorporated as a functional additive in perovskite solar cells. Its inclusion significantly boosted the power conversion efficiency from 20.60% to 22.61%, suggesting its crucial role in optimizing charge transport within the active layer and notably improved device‐to‐device reproducibility. Our work provides insights into the development of bottom‐up synthesis of novel non‐planar nanographenes incorporating five‐ and eight‐membered rings and their potential applications in optoelectronic devices.

## Author Contributions

**Qiang Huang**: writing – original draft, writing – review and editing, data curation, methodology, investigation, conceptualization, formal analysis, validation, visualization. **Yubin Fu**: data curation, conceptualization. **Zongbao Zhang**: methodology, data curation, investigation. **Jinjin Ding**: data curation, investigation, visualization. **Mengyuan Yu**: writing – original draft. **Xinyang Ge**: visualization. **Boris Borrisov**: formal analysis. **Hartmut Komber**: formal analysis, data curation, writing – review and editing. **Zhen‐Lin Qiu**: data curation. **Yana Vaynzof**: supervision, resources. **Ji Ma**: supervision, writing – review and editing, funding acquisition. **Xinliang Feng**: supervision, resources, funding acquisition, writing – review and editing, conceptualization, project administration.

## Conflicts of Interest

The authors declare no conflicts of interest.

## Supporting information




**Supporting File**: anie73005‐sup‐0001‐SuppMat.docx.

## Data Availability

The data that supports the findings of this study are available in the supplementary material of this article.
