# Supplementary material for: A Nonbenzenoid Nanographene Horse Saddle With Four Pentagon–Octagon Pairs
Source: Angew Chem Int Ed Engl. 2026 Jun 4;65(30):e2412282. doi: 10.1002/anie.2412282 (PMC13383261; doi:10.1002/anie.2412282)
Supplement: Supplementary file 1 — Supporting File: anie73005‐sup‐0001‐SuppMat.docx. [file ANIE-65-e2412282-s001.docx]

*Supporting Information*

**A Nonbenzenoid Nanographene Horse Saddle with Four Pentagon-Octagon Pairs**

Qiang Huang*, Yubin Fu, Zongbao Zhang, Jinjin Ding, Mengyuan Yu, Xinyang Ge, Boris Borrisov, Hartmut komber, Zhen-lin Qiu, Yana Vaynzof, Ji Ma*, and Xinliang Feng*

1. **General Information**
2. **Synthesis Procedures and Geometrical Configuration**
3. **HR-MS Characterization of 10 and HN1**
4. **NMR Spectra for New Compounds and Geometrical Configuration**
5. **Optoelectronic Devices**
6. **References**

**1. General Information**

All chemicals and anhydrous solvents are purchased from commercial suppliers or synthesized according to the literatures. UV-*vis* absorption spectra were recorded on an Agilent Cary 5000 UV-VIS-NIR spectrophotometer by using 10 mm optical-path quartz cell at room temperature. Fluorescence spectra were recorded at room temperature on a PerkinElmer. The NMR spectra were recorded on a Bruker BioSpin (^1^H 400 MHz, ^13^C 100 MHz) spectrometer and chemical shifts are expressed in parts per million (ppm) relative to CHCl_3_ (δ = 7.26 ppm)/ CD_2_Cl_2_ (δ = 5.30 ppm) for ^1^H NMR and CDCl_3_ (δ = 77.00 ppm) / CD_2_Cl_2_ (δ = 54.00 ppm) for ^13^C NMR. Use MALDI-TOF-MS technology for high resolution mass spectrometry (HR-MS) analysis, Data are reported as follows: chemical shift, integrals, multiplicity (s = singlet, d = doublet, m = multiplet), coupling constant (*J*, Hz). Silica gel GF254 pre-coated plates were used for preparation of thin layer chromatography (PTLC), and chromatographic separation was performed on silica gel (200~300 mesh). All air-sensitive reactions are carried out in a nitrogen or argon atmosphere. Cyclic voltammetry (CV) and Square-wave voltammetry (SWV) measurements were carried out at room temperature on PARSTAT4000 potentiostat using a three-electrode system in degassed solution.

**2. Synthesis Procedures and Geometrical Configuration**

**Synthesis** **2-(2-bromonaphthalen-1-yl)acetic acid** **(7).**

2-(2-bromonaphthalen-1-yl)acetic acid (**7**) were prepared according to the published procedures.^1^

**Synthesis of compound** **3-bromoacenaphthylen-1(2H)-one (9).** 2.66 g (10.00 mmol) of 2-(2-bromonaphthalen-1-yl)acetic acid **7** were dissolved in 8 mL of thionylchloride and reflux for 1 h under nitrogen atmosphere. The excess of thionylchloride was removed under vacuum to afford 2-(2-bromonaphthalen-1-yl)acetyl chloride **8**. The resulting oil was dissolved in 10 mL of CH_2_Cl_2_ and cooled down to 0 °C (nitrogen atmosphere), before 2.66 g (20 mmol) of AlCl_3_ was slowly added. The mixture was stirred for 30 min at 0 °C and then heated to reflux for 30 min. After cooling to room temperature, the mixture was slowly poured to a suspension of 100 g of ice and 10 mL of hydrochloric acid. After warming up to room temperature the product was extracted with DCM (3 x 50 mL). Crude product was purified by flash column chromatography on silica, using hexane/CH_2_Cl_2_ (1:1) as an eluent to afford **9** as white solid (1.71 g, 69%). ^1^H NMR (400 MHz; CDCl_3_): δ 8.07 (d, 8.4 Hz, 1H), 7.97 (d, 7.4 Hz, 1H), 7.73 (d, 8.7 Hz, 1H), 7.72 (t, 1H), 7.66 (d, 8.7 Hz, 1H), 3.78 ppm (s, 2H). ^13^C NMR (100 MHz; CDCl_3_): δ 200.7, 143.6, 135.7, 135.0, 131.8, 131.6, 129.5, 128.2, 126.1, 122.7, 115.9, 43.0 ppm.

**Synthesis of compound 3,9,15,21-tetrabromocycloocta[1,2-a:3,4-a':5,6-a'':7,8-a''']tetraacenaphthylene (10).** Into a flame-dried 200 mL three-necked round-bottomed flask, fitted with a condenser and a nitrogen inlet, was placed 20 mL of o-dichlorobenzene. To this was added 0.68 mL (4.65 mmol) of titanium tetrachloride by syringe, and the resulting yellow solution was heated to reflux. A solution of 247.09 mg (1 mmol) of 3-bromoacenaphthylen-1(*2H)*-one **9** in 20 mL of *o*-dichlorobenzene was then added dropwise. The reaction mixture was maintained at 180 °C for an additional 1 h. The resulting black solution was allowed to cool to room temperature and was then poured over concentrated hydrochloric acid/ice to quench the reaction. Dilution with 200-300 mL of methylene chloride gave a dark red organic layer. Evaporation of the solvents under reduced pressure gave a dark red/ brown solid. The residue was purified by silica gel column chromatography (CH_2_Cl_2_/hexane = 1/4) to give 10 as a brown solid in 18% (41.24 mg, 45 μmol). HRMS (MALDI-TOF) m/z calcd. for C_48_H_20_Br_4_ [M]^+^: 915.8258, found: 915.8255. ^1^H and ^13^C NMR data: The ^1^H and ^13^C NMR spectra of **10** are very complex and characterised by a large number of overlapping signals. This is due to the occurrence of different stereoisomers resulting from different arrangements (up or down) of the five-membered rings on the central eight-membered ring. The relevant bonds are highlighted in color in the formula. Therefore, instead of listing chemical shifts, Figures S7 and S8 show the ^1^H NMR spectrum and the ^13^C NMR spectrum (including the DEPT135 NMR spectrum) as a reference for fingerprint comparison.

The HSQC spectrum (Figure S9) allows the identification of five signal regions for the five different protons 1-3, 4 and 5. Further assignments are possible using the HSQC-TOCSY spectrum (Figure S10) and the multiplicity (only H_2_ leads to a triplet). A more detailed signal assignment failed due to the complexity of the spectra, even when using 2D NMR methods.

**Synthesis of compound HN1.**

Tetrabromocyclooctatetraacenaphthylene **10** (50.00 mg, 54.57 µmol), 1,2-bis(4-(tert-butyl)phenyl)ethyne (69.74 mg, 240.09 µmol), AgOAc (40.07 mg, 240.09 µmol), Pd(OAc)_2_ (1.84 mg, 8.19 µmol), and P(4-ClC_6_H_4_)_3_ (2.99 mg, 8.19 µmol) were dissolved in DCE (10 mL) added to the Schlenk tube under N_2_ atmosphere and stirred at 80 °C for 36 h. The reaction mixture was cooled to room temperature, and then concentrated under reduced pressure. The residue was purified by silica gel column chromatography (CH_2_Cl_2_/hexane = 1/3) to give **HN1** as a dark brown solid in 32% (25.6 mg, 17.46 μmol). ^1^H NMR (CD_2_Cl_2_): δ 8.45 (d, 7.0 Hz, 2H; 1), 7.66 (d, 7.4 Hz, 2H; 3), 7.64 (d, 8.7 Hz, 2H; 4), 7.62 (t, 2H; 2), 7.50 (d, 8.7 Hz, 2H; 5), 7.45 (2d, 4H; 7, 8), 7.35 (d, 8.3 Hz, 4H; 10), 7.32 (d, 8.4 Hz, 2H; 9), 7.30 (d, 8.6 Hz, 2H; 6), 7.26 (d, 8.4 Hz, 4H; 13), 7.13-7.09 (8H; 11, 14), 7.07 (d, 8.5 Hz, 4H; 16), 7.02 (d, 8.5 Hz, 4H; 17), 1.17 (s, 18H; 15), 1.15 (s, 18H; 12), 1.13 ppm (s, 18H; 18). ^13^C NMR (CD_2_Cl_2_): δ 149.5, 144.4, 143.4, 141.2, 139.1, 131.0 (6), 130.9 (9), 130.4 (13), 130.1 (10, 16), 130.0 (5), 129.4, 127.5 (2), 125.4 (4), 125.1 (3), 125.0 (7, 8); 124.9 (11, 14), 124.6 (17), 120.7 (1), 34.5 (C of tBu), 31.4 (15), 31.3 (12), 31.1 (18). The assignment of CH and CH_3_ group signals is based on the HSQC spectrum. HRMS (MALDI-TOF) m/z calcd. for C_114_H_96_ [M]^+^: 1465.7542, found: 1465.7546.


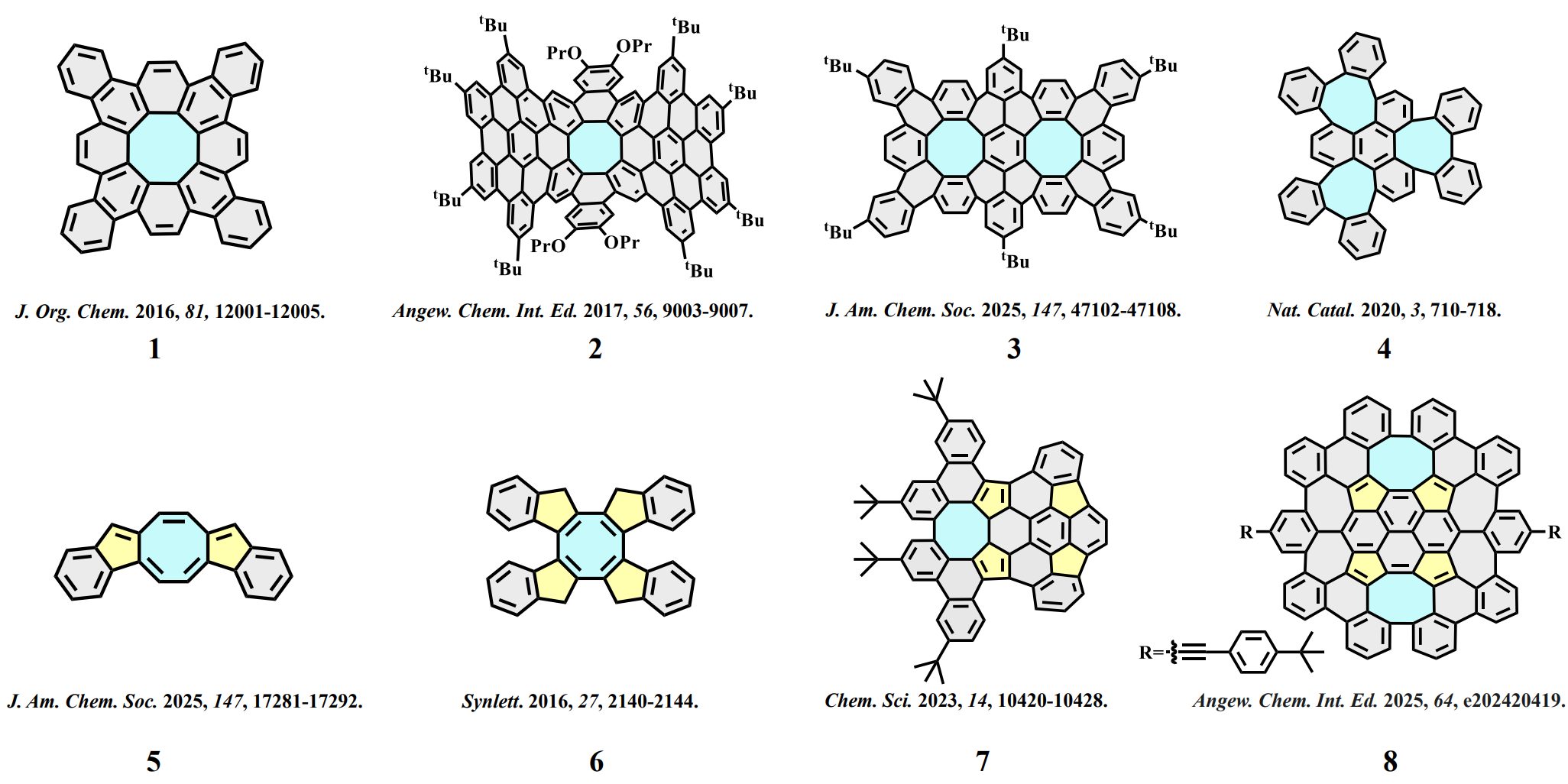


**Figure S1**. Recent examples of octagon and pentagon-octagon embedded NGs.^2^

**3. HR-MS characterization of 10 and HM**


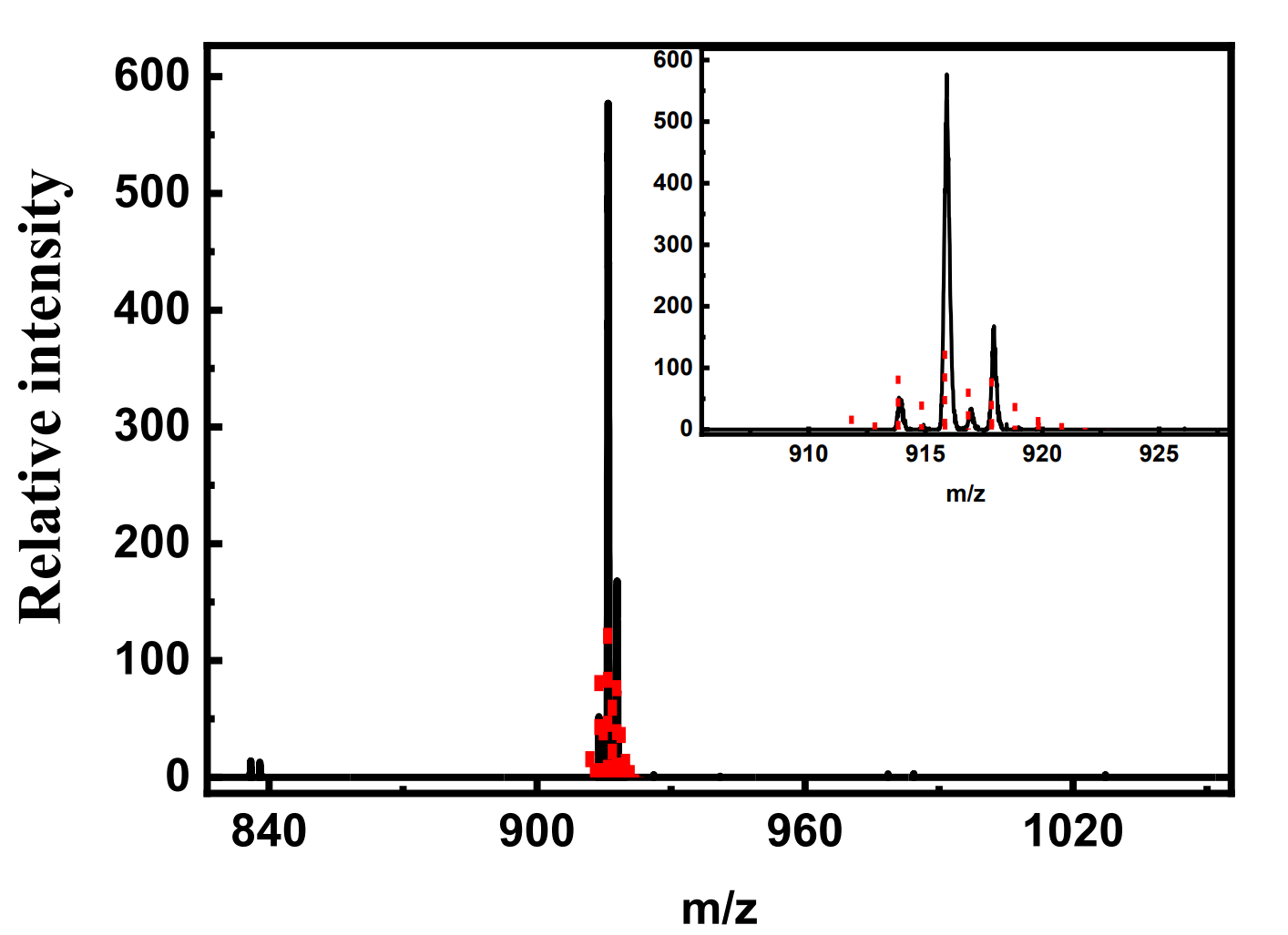


**Figure S2.** MALDI-TOF-MS spectrum (black) and simulated data (red) for **10.**


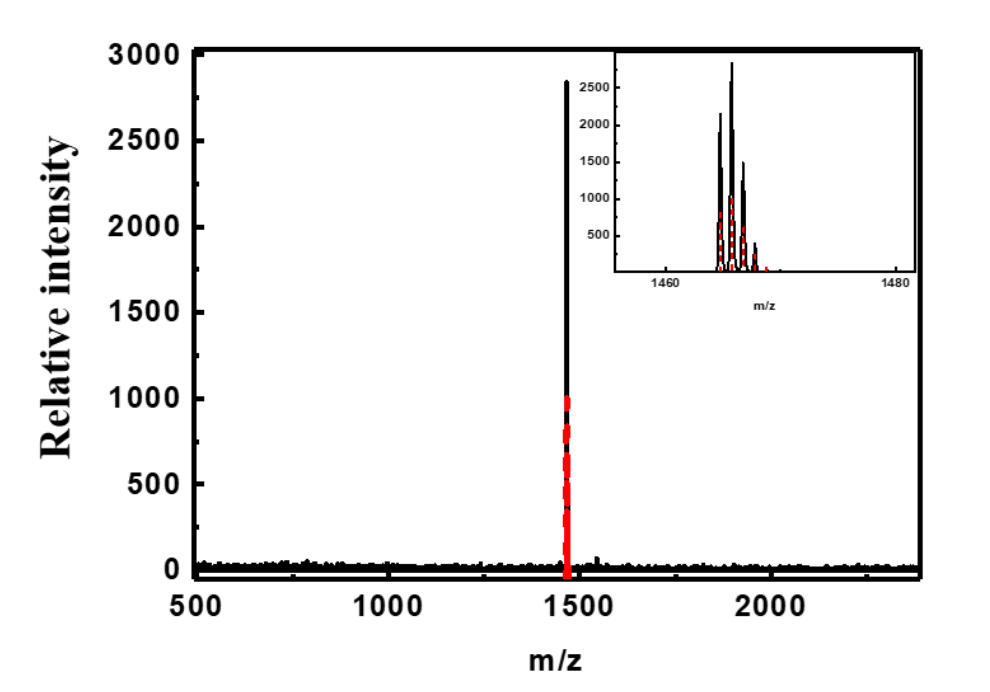


**Figure S3.** MALDI-TOF-MS spectrum (black) and simulated data (red) for **HN1**.

**4.**  **^1^H NMR and ^13^C NMR copies for new compounds**


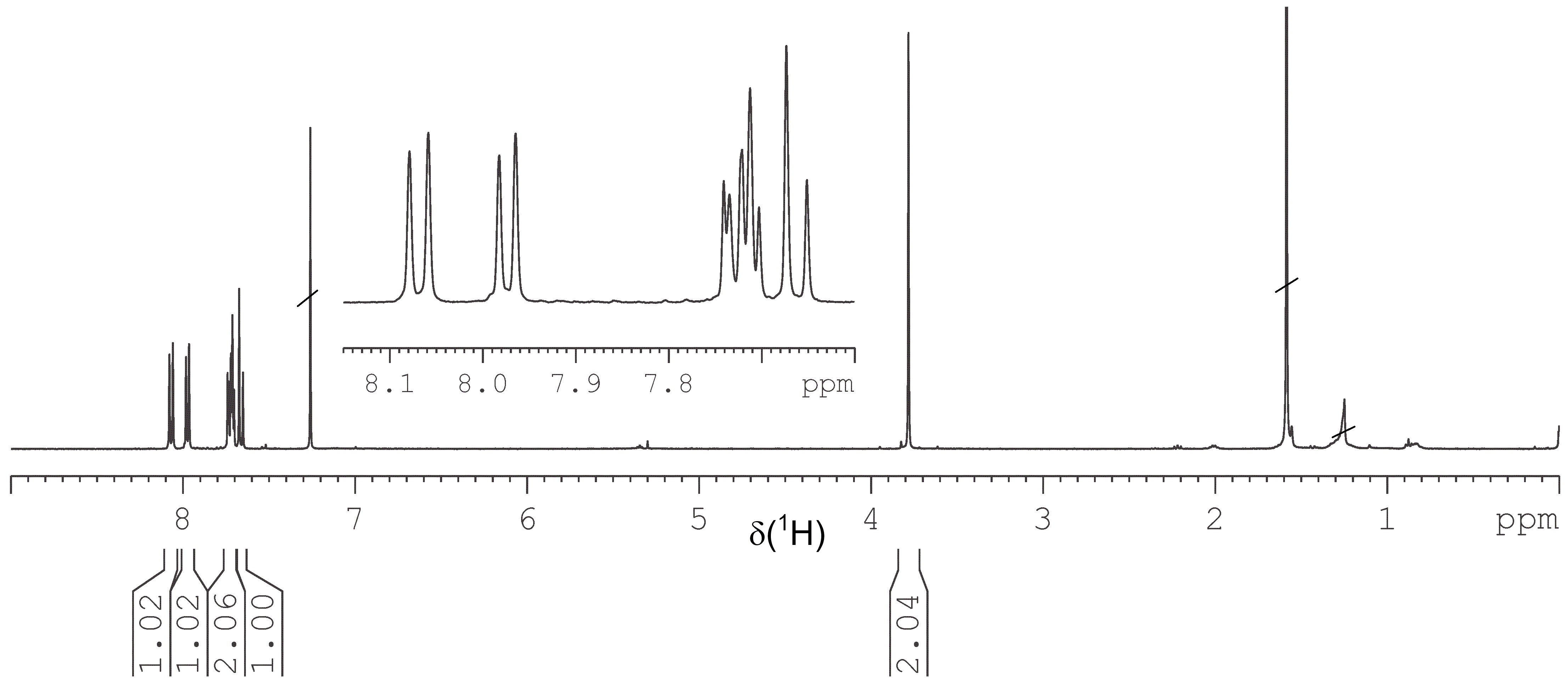


**Figure S4.** ^1^H NMR spectrum (400 MHz) of **9** (solvent: CDCl_3_).


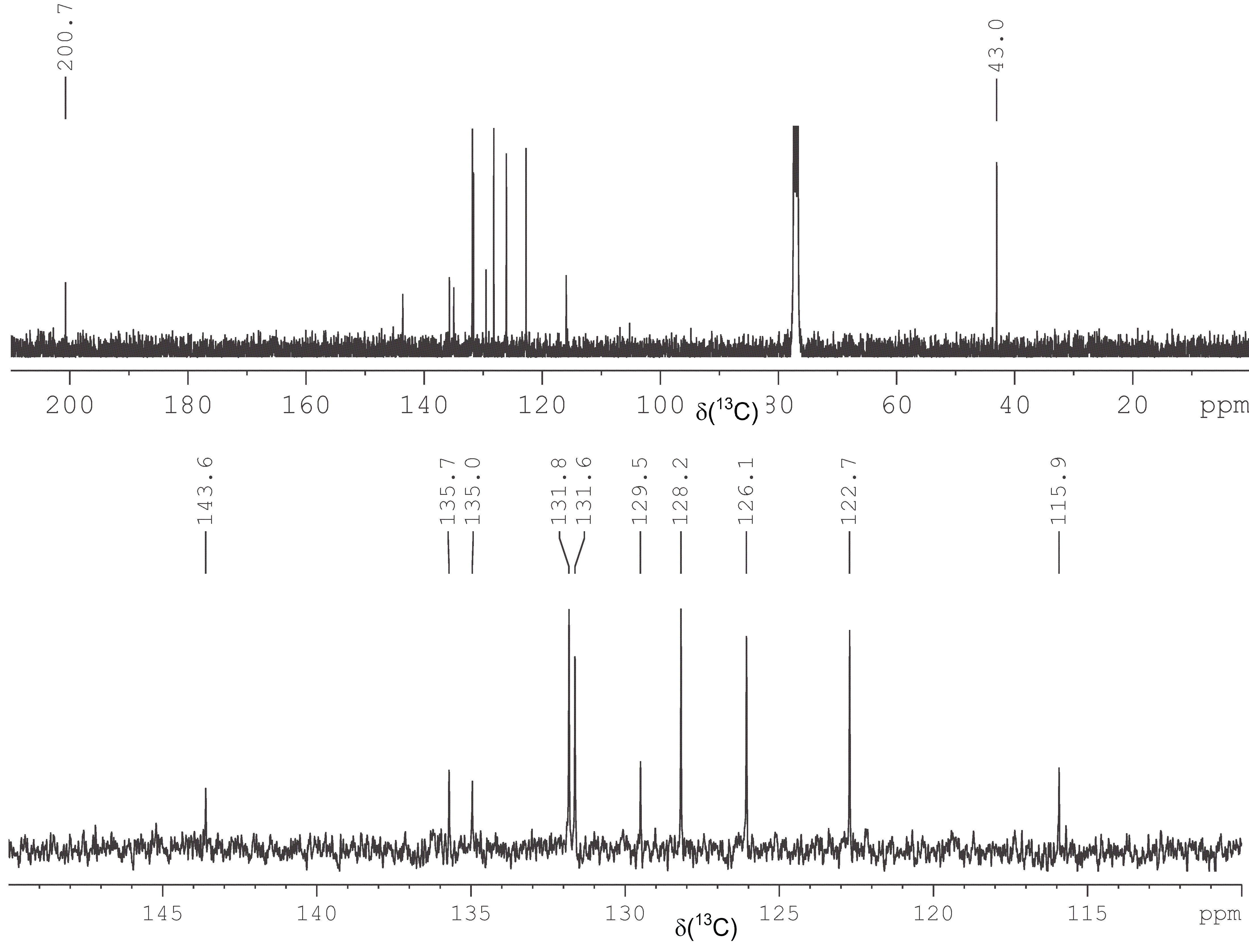


**Figure S5.** ^13^C NMR spectrum of **9**.


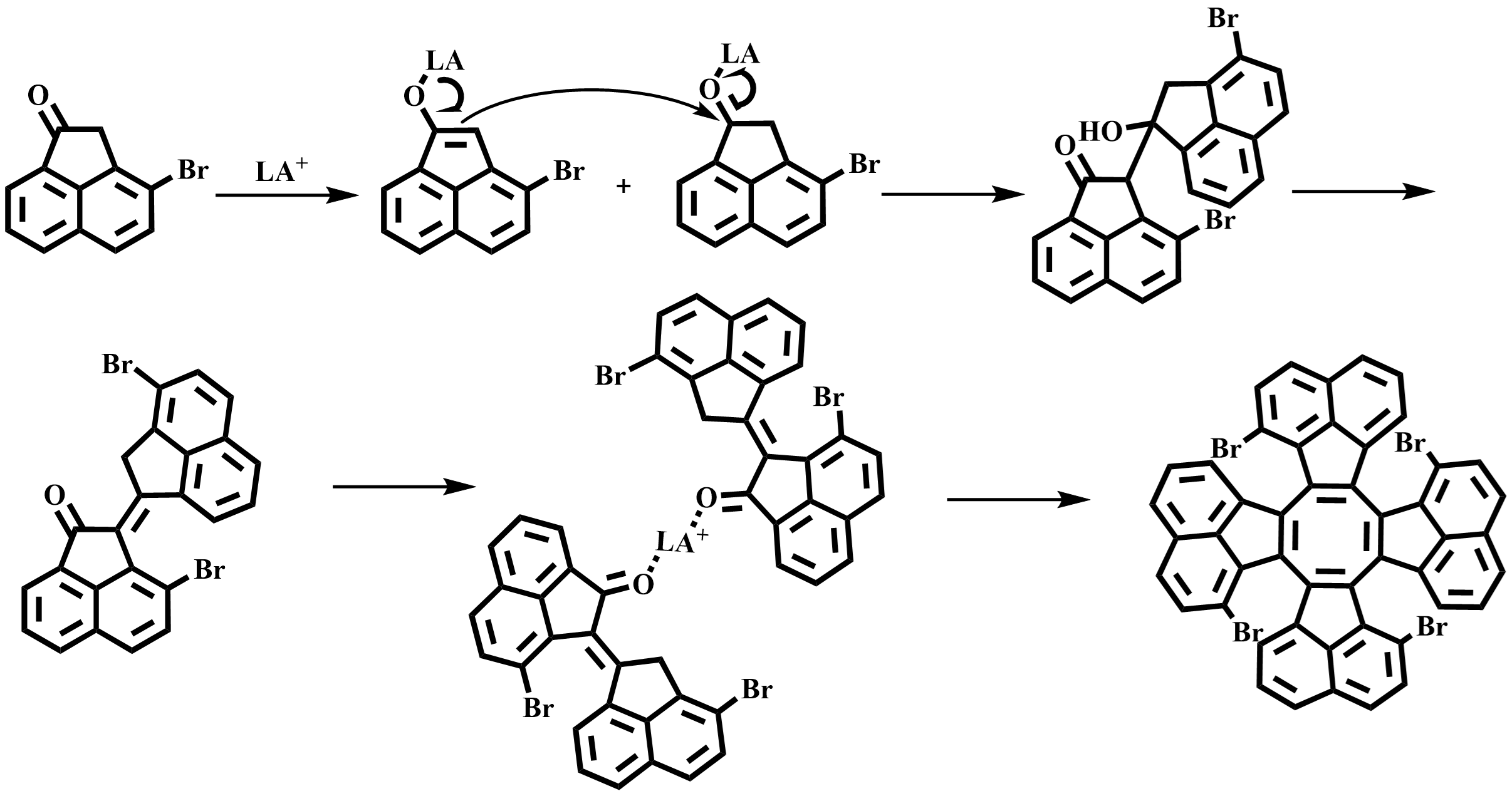


**Figure S6.** The reaction mechanism of Aldol cyclotetramerization.

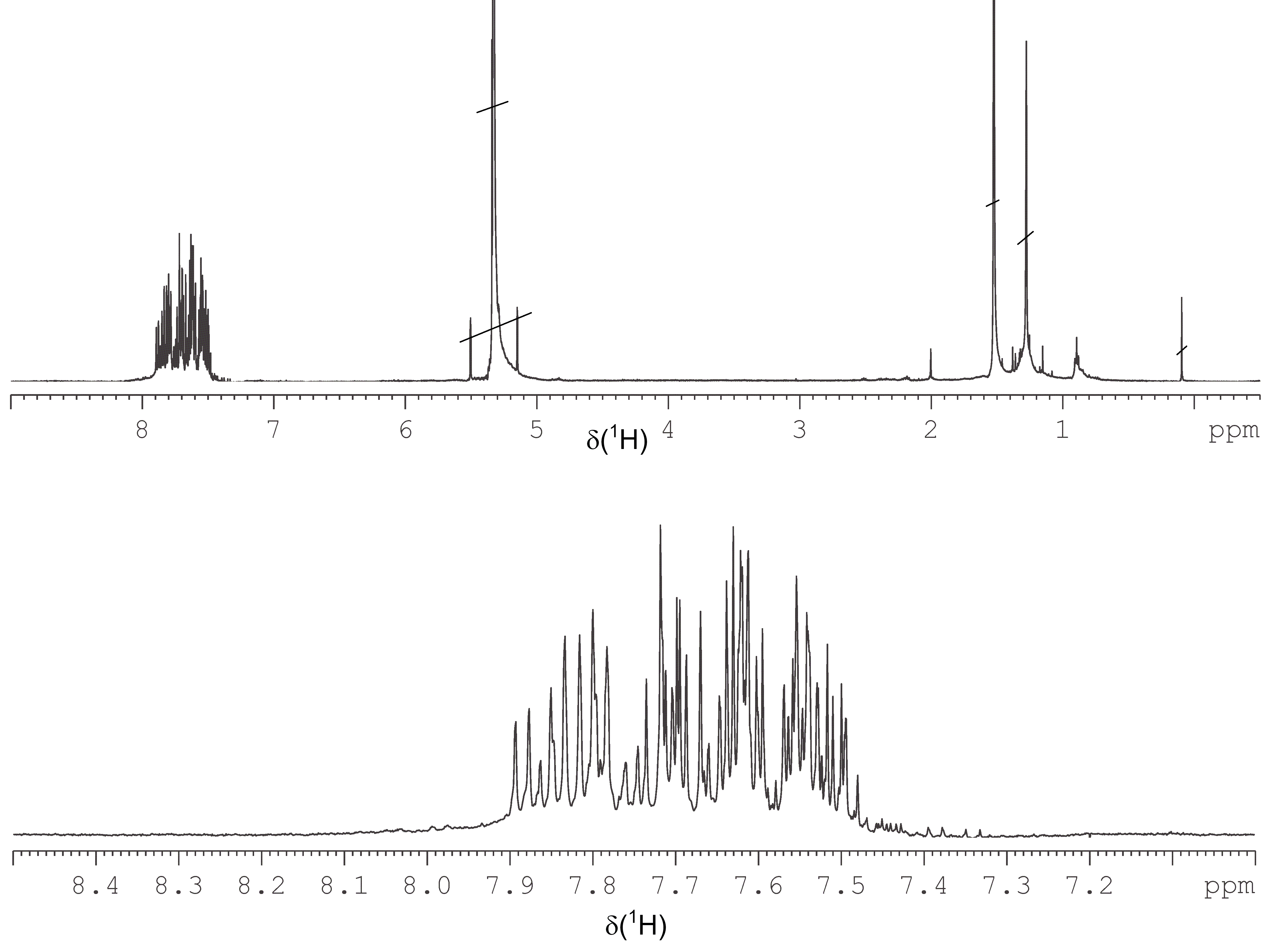


**Figure S7.** ^1^H NMR spectrum of **10** (solvent: CD_2_Cl_2_).


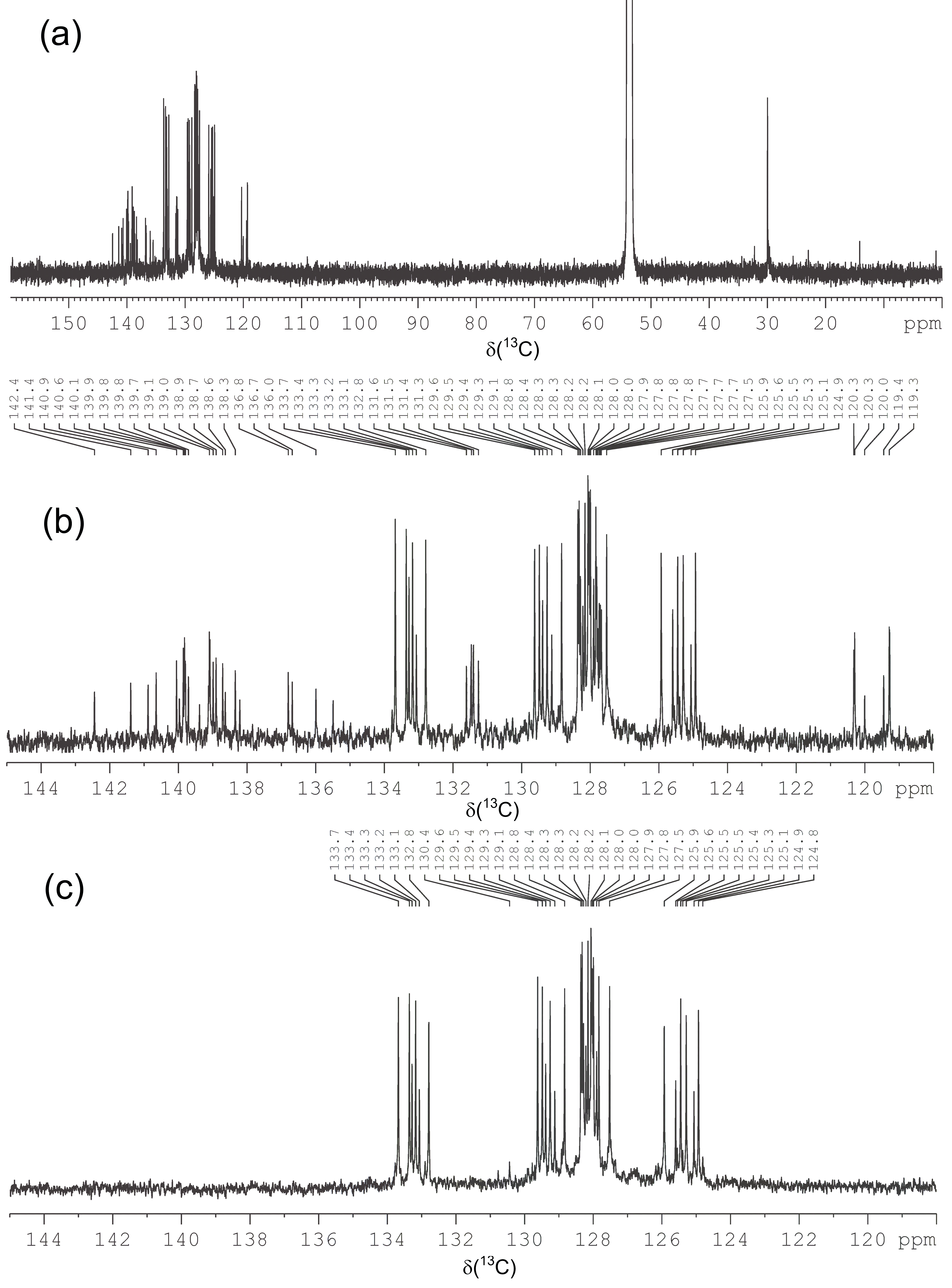


**Figure S8.** ^13^C NMR spectrum of **10** (solvent: CD_2_Cl_2_). (a) overview, (b) region of aromatic carbons and (c) DEPT135 spectrum only showing signals of aromatic CH carbons.


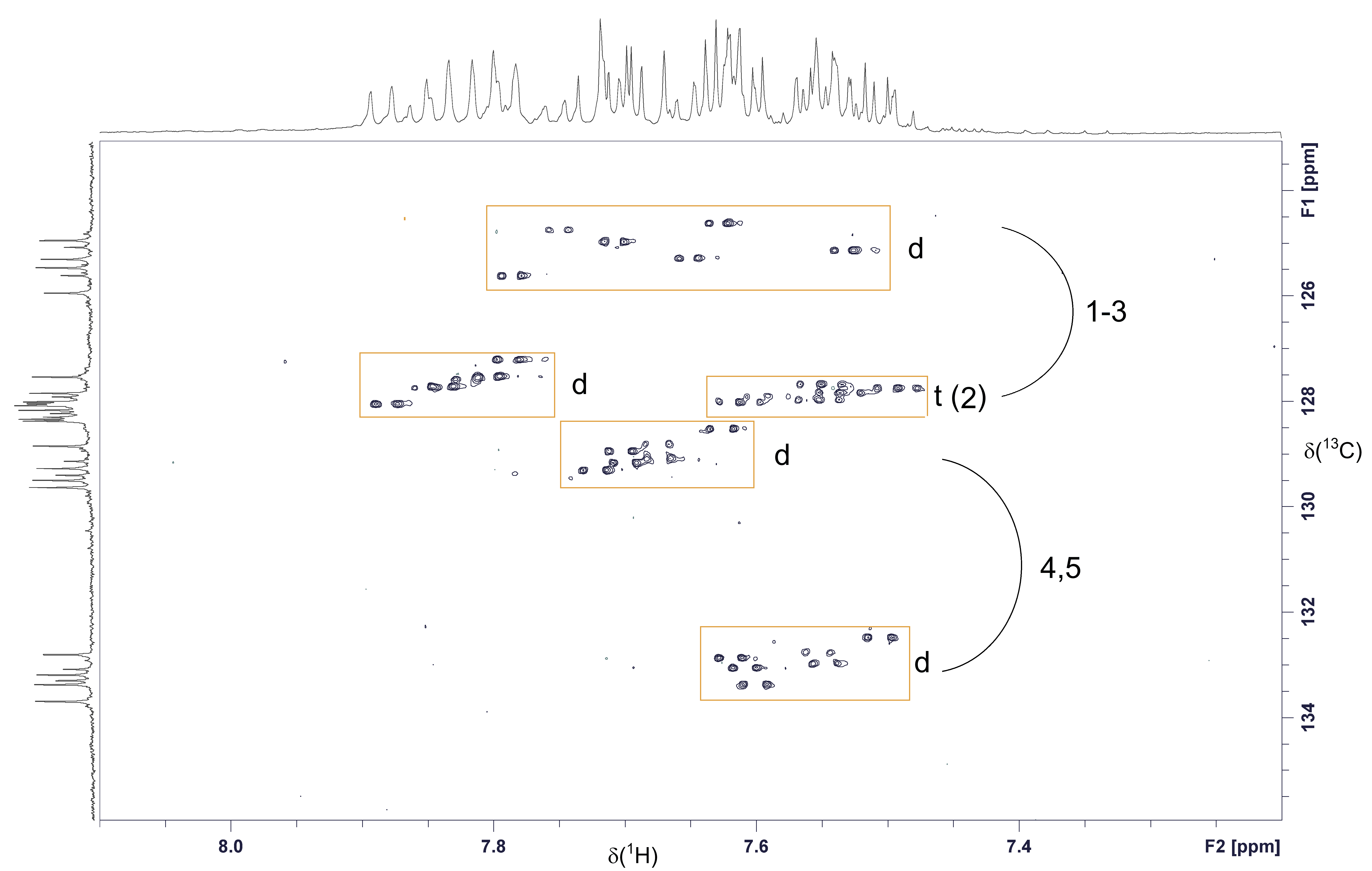


**Figure S9.** HSQC spectrum of **10** (solvent: CD_2_Cl_2_). The F1 axis shows the DEPT135 spectrum. Five different signal ranges can be distinguished, corresponding to the number of different CH carbon atoms. The multiplicities of the corresponding proton signals are indicated as d (doublet) and t (triplet).


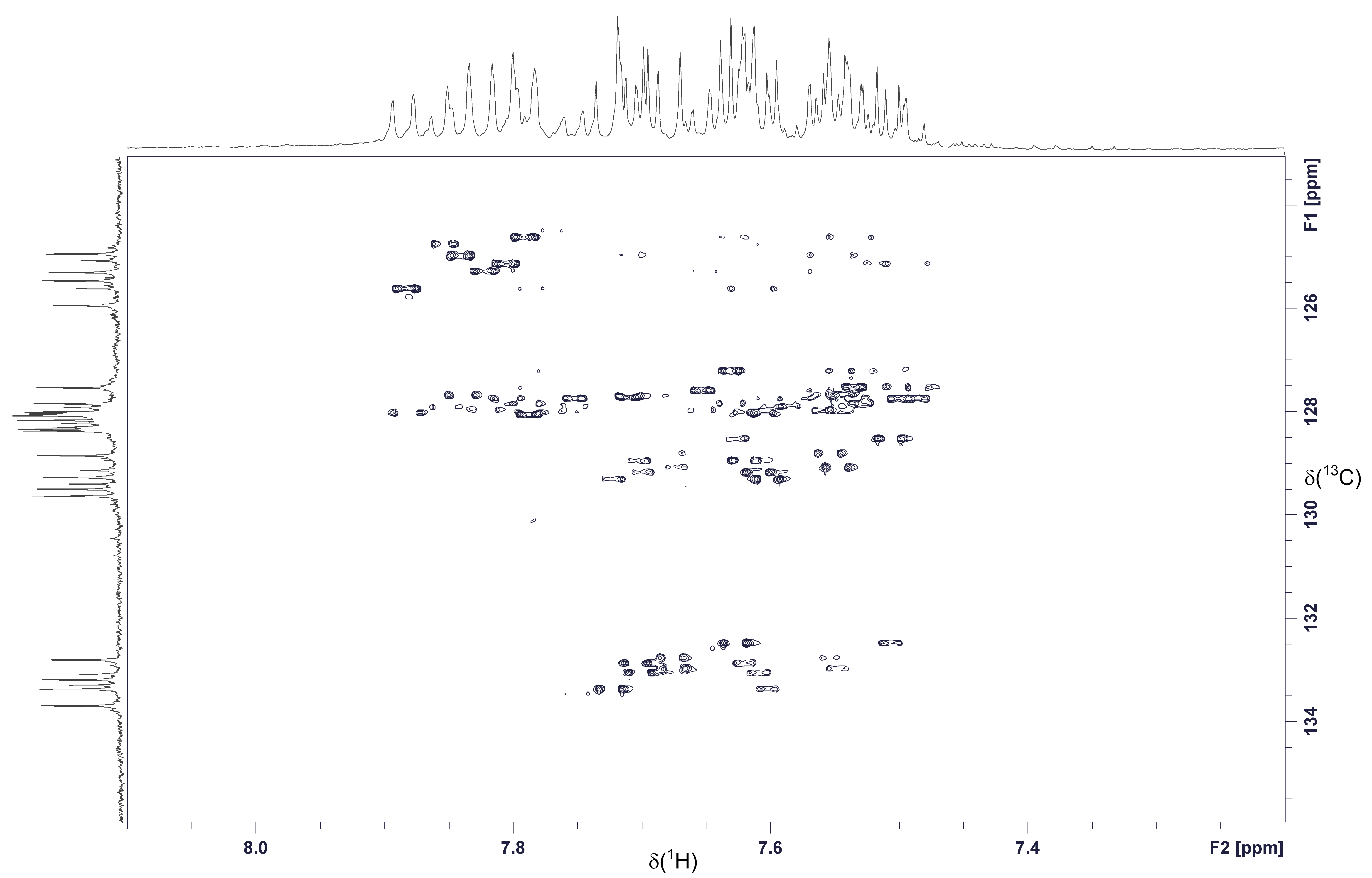


**Figure S10.** HSQC-TOCSY spectrum of **10** (solvent: CD_2_Cl_2_). The F1 axis shows the DEPT135 spectrum.


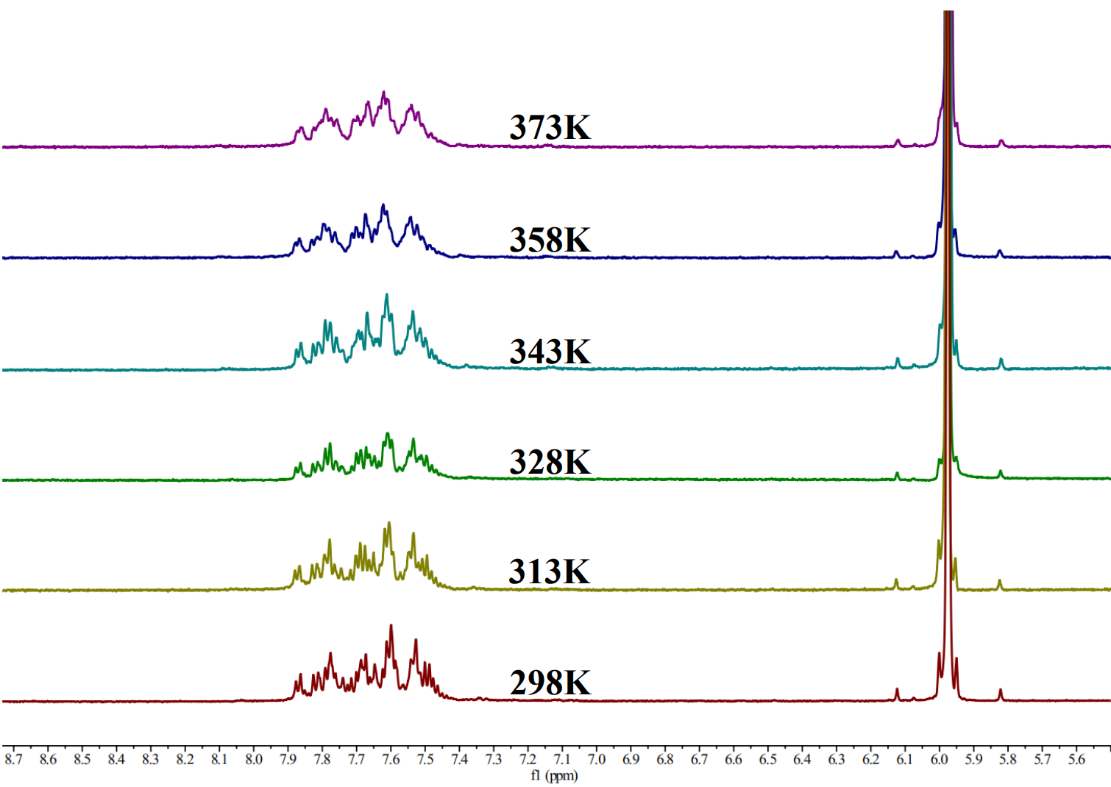


**Figure S11.** Variable-Temperature NMR (*VT*-NMR) spectrum of **10** in C_2_Cl_4_D_2_.


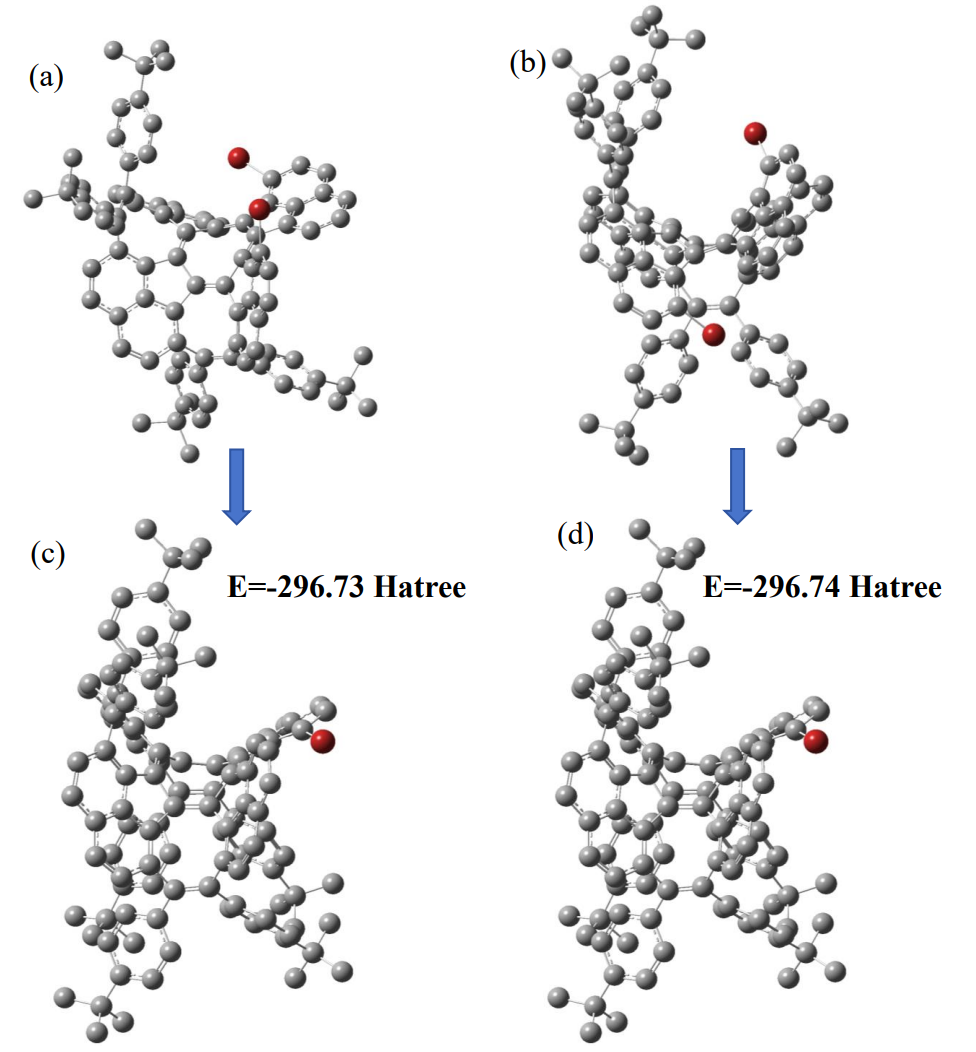


**Figure S12.** Calculated optimized geometries of the two stereoisomers (a, b) and their annulation products (c, d). (a) Two Br atoms up; (b) One Br up, one down; (c) Product from (a); (d) Product from (b). Both pathways converge to the same product geometry and energy.


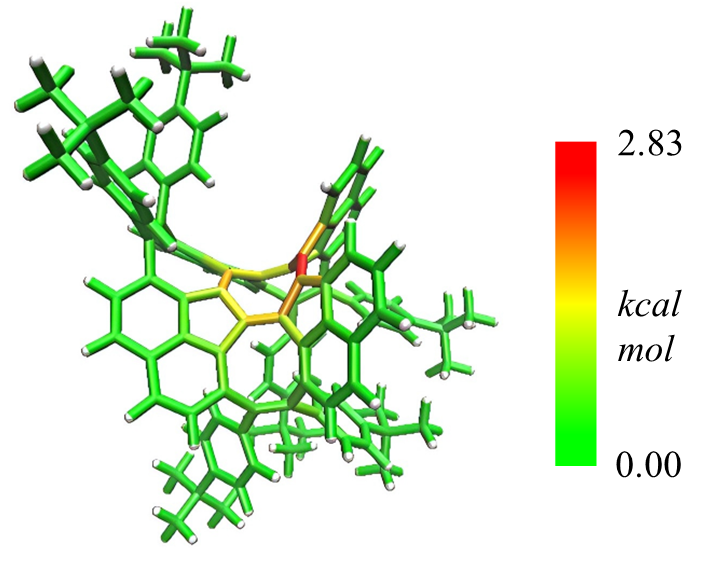


**Figure S13.** Strain energy present in **HN1**.


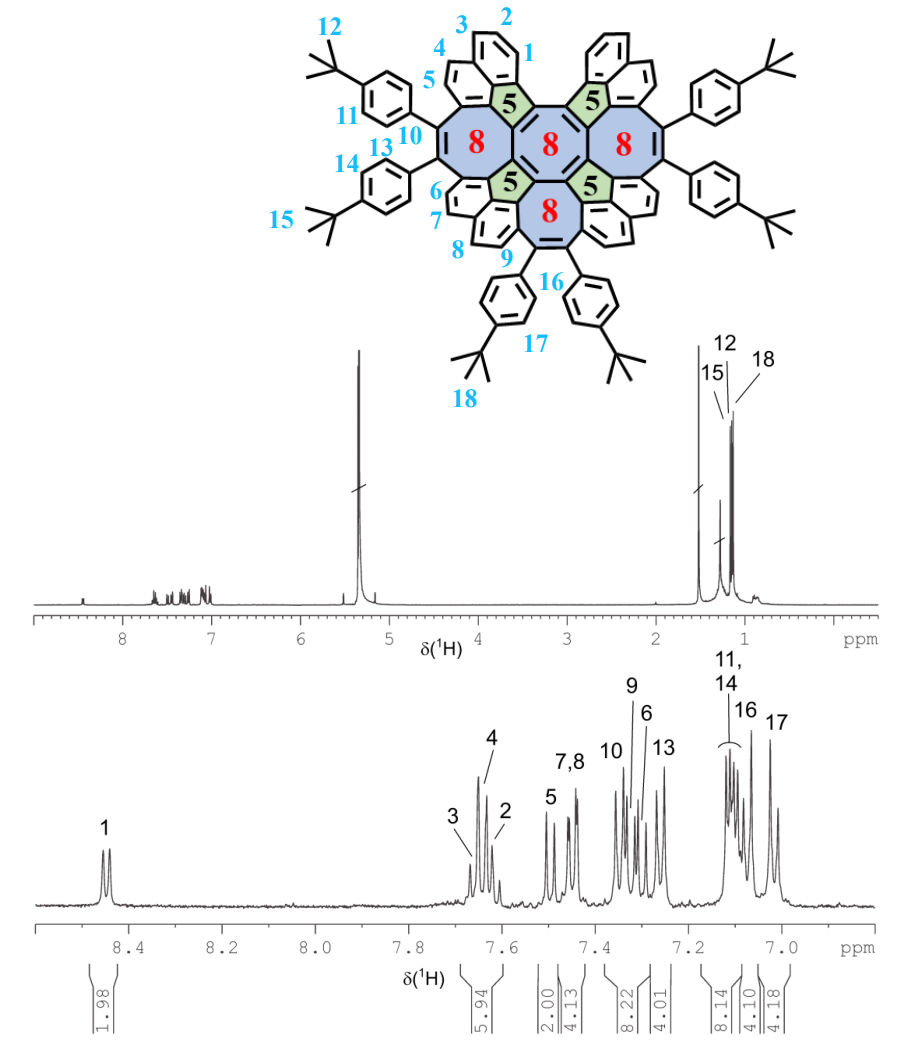


**Figure S14.** ^1^H NMR spectrum of **HN1** (solvent: CD_2_Cl_2_).


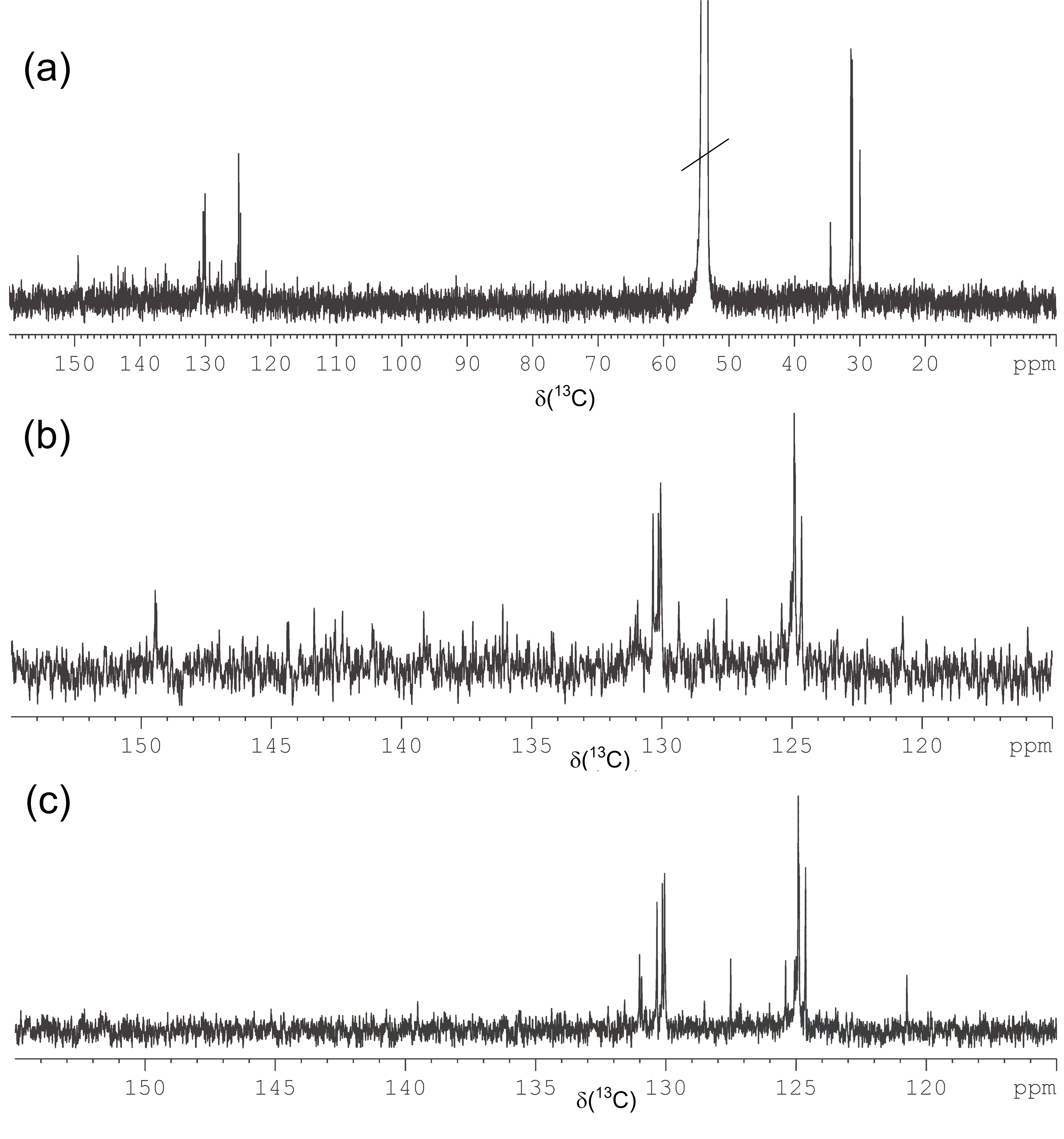


**Figure S15.** ^13^C NMR spectrum of **HN1** (solvent: CD_2_Cl_2_). (a) overview, (b) region of aromatic carbons and (c) DEPT135 spectrum (region of aromatic carbons).


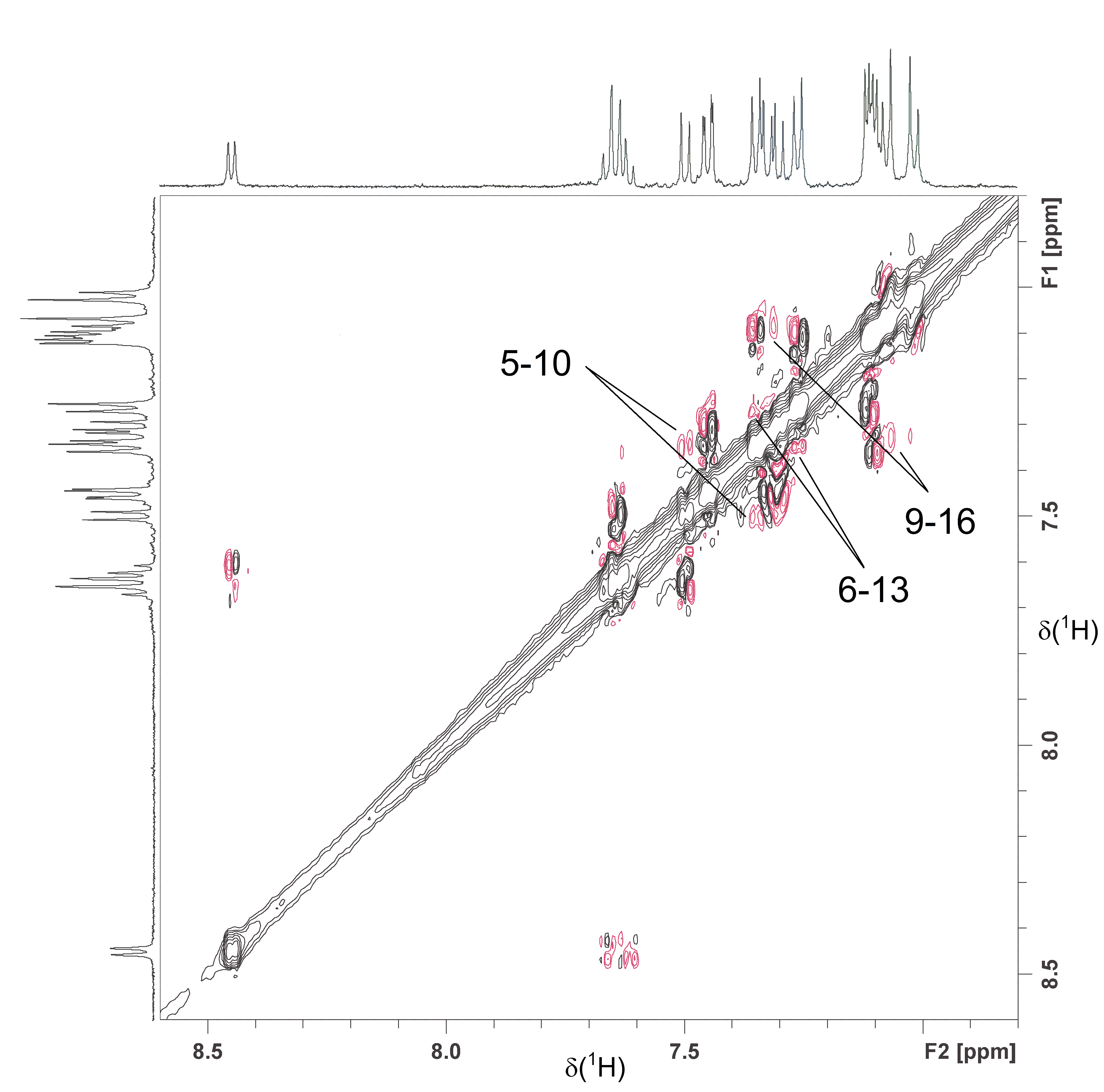


**Figure S16.** ROESY spectrum of **HN1** (solvent: CD_2_Cl_2_). The figure shows the correlations between aromatic protons. Unfortunately, TOCSY cross peaks appear as intense artefacts. However, the ROESY correlations important for signal assignment (marked in the figure) can be easily identified.


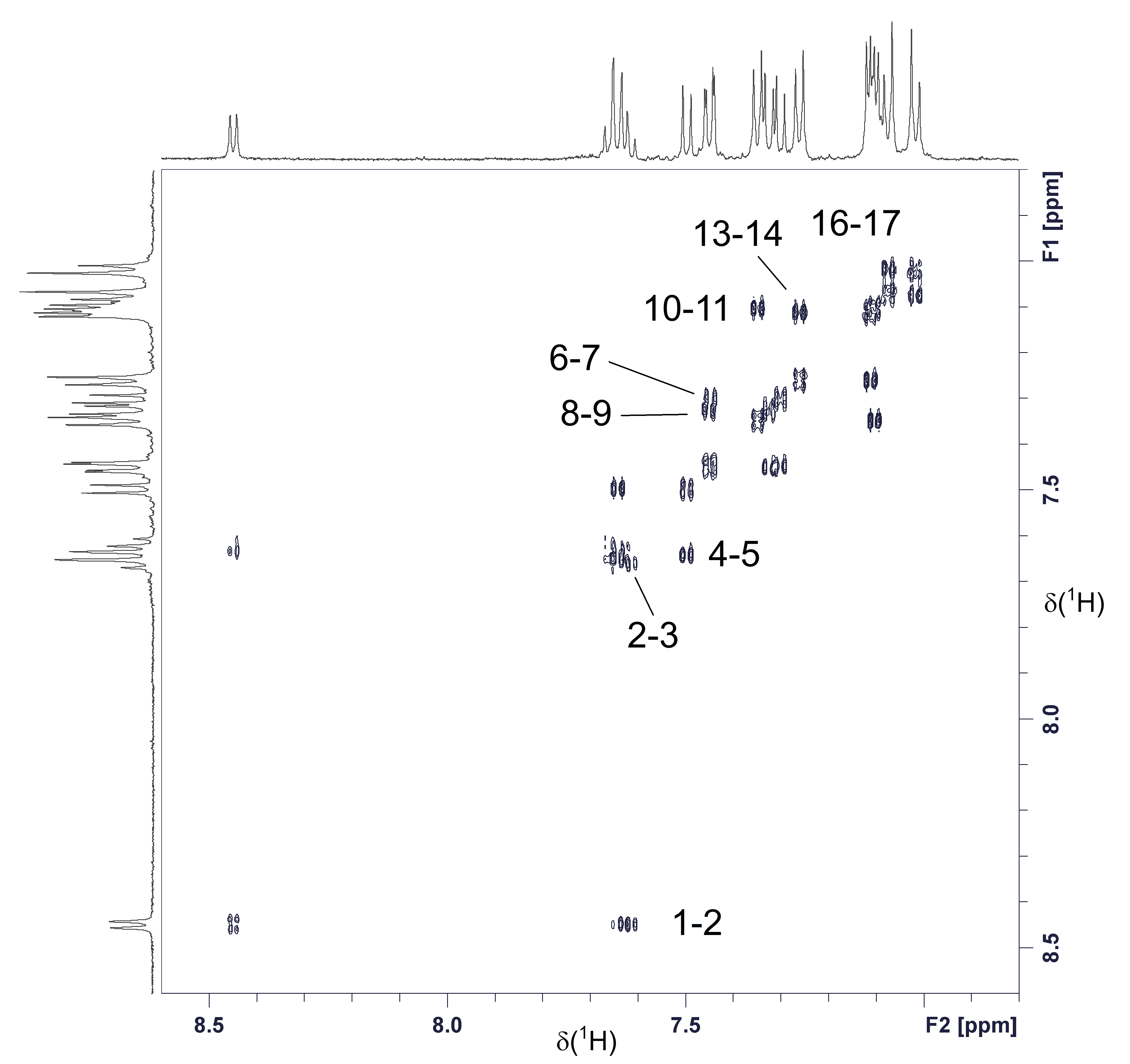


**Figure S17.** COSY spectrum of **HN1** (solvent: CD_2_Cl_2_).


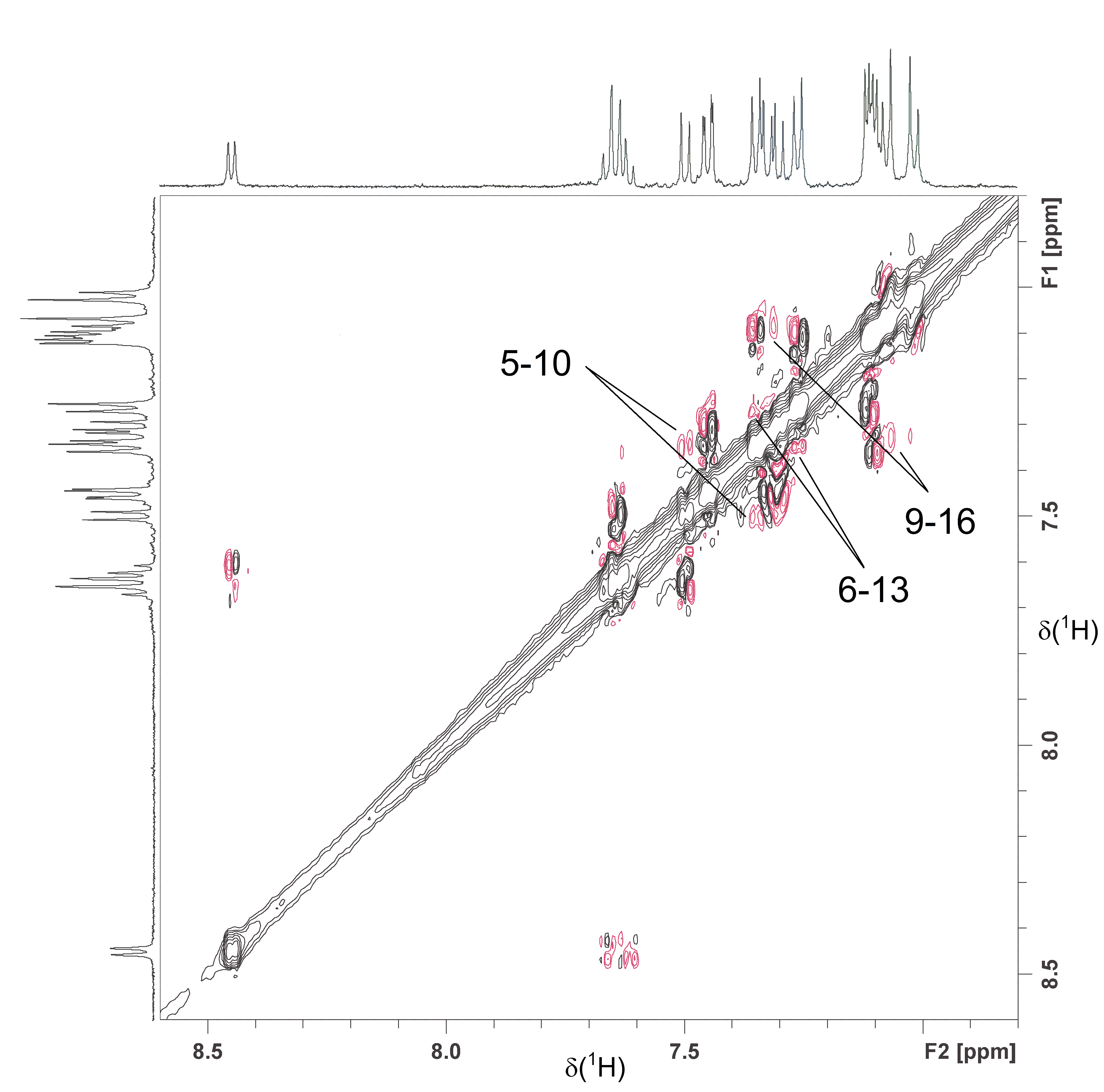


**Figure S18.** ROESY spectrum of **HN1** (solvent: CD_2_Cl_2_). The figure shows the correlations between aromatic protons. Unfortunately, TOCSY cross peaks appear as intense artefacts. However, the ROESY correlations important for signal assignment (marked in the figure) can be easily identified.


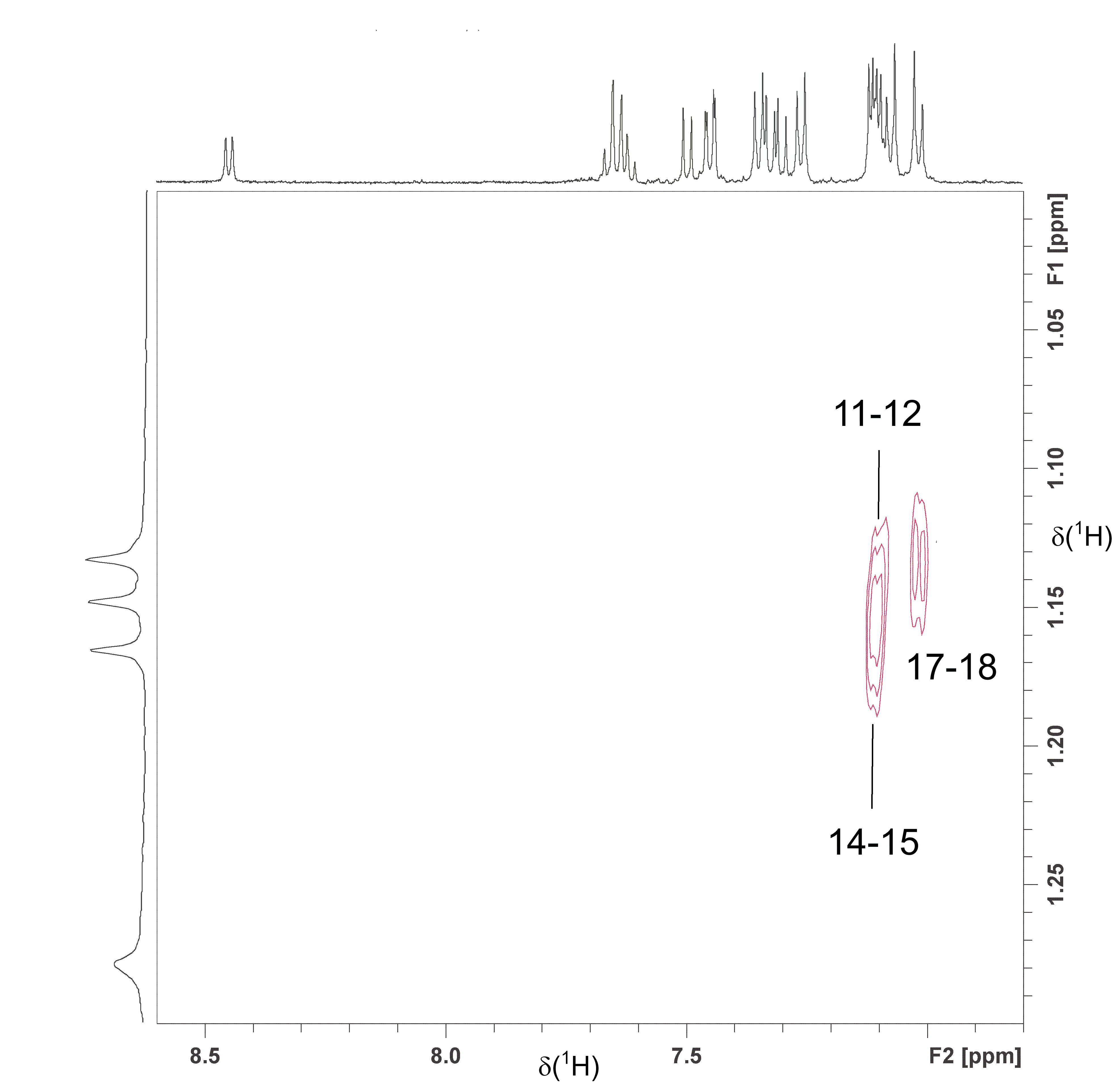


**Figure S19.** ROESY spectrum of **HN1** (solvent: CD_2_Cl_2_). The figure shows the correlations between aromatic protons and methyl group protons.


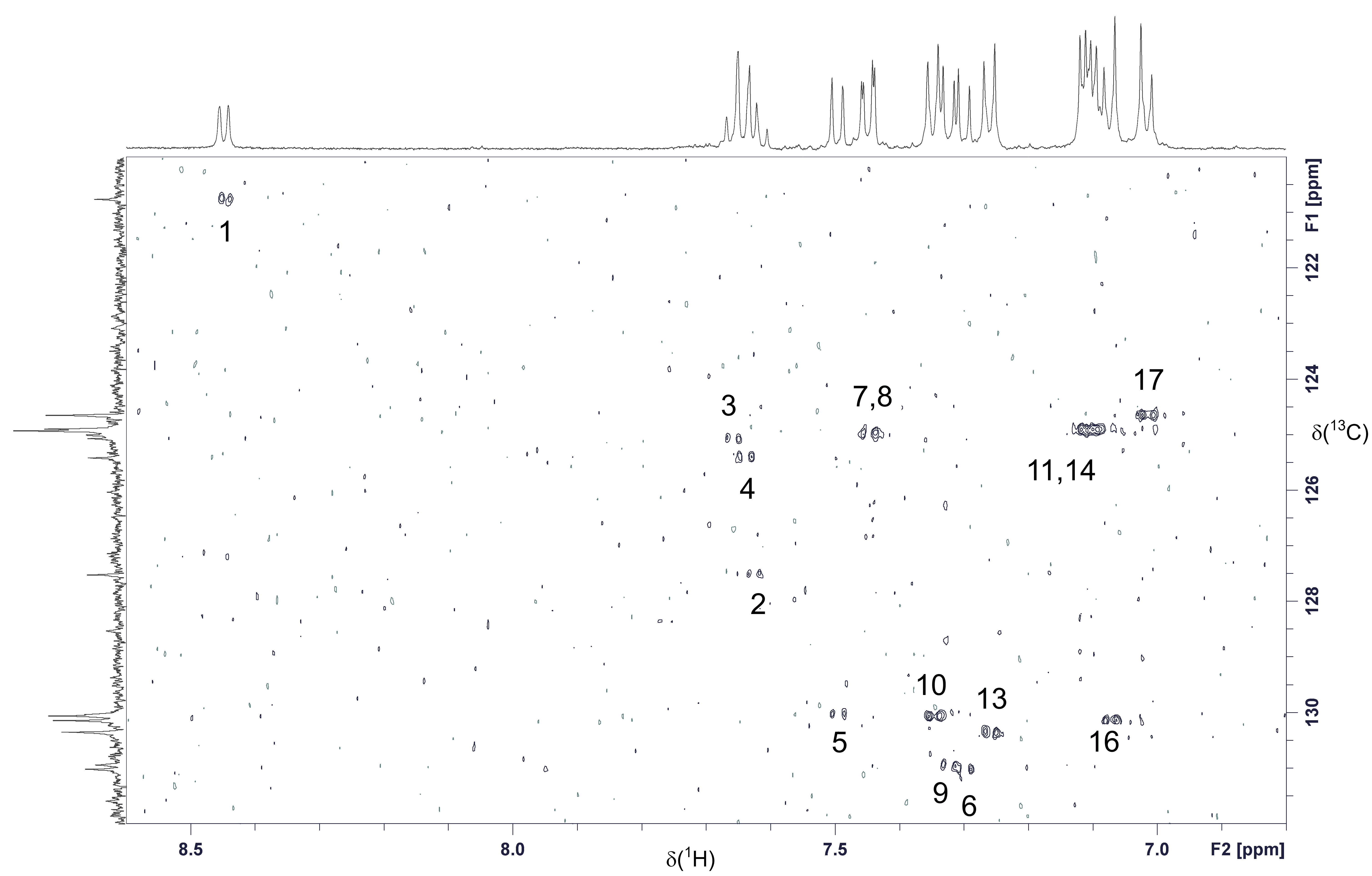


**Figure S20.** HSQC spectrum of **HN1** (solvent: CD_2_Cl_2_). The figure shows the correlations of the aromatic CH groups. The F1 axis shows the DEPT135 spectrum.

| **Table S1.** Crystal data, data collection and refinement of **NH1**. | |
| --- | --- |
| Complex | **NH1@** **chlorobenzene** |
| Formula | C_126_H_106_Cl_2_ |
| Formula weight | 1691.00 |
| Temperature/K | 100 |
| CCDC number | [2523651](https://www.ccdc.cam.ac.uk/mystructures/viewinaccessstructures/92bb3ec2-68f3-f011-96dd-00505695281c) |
| Crystal system | monoclinic |
| Space group | P 21/n |
| a/Å | 12.748(3) |
| b/Å | 41.316(8) |
| c/Å | 19.112(4) |
| α/deg | 90 |
| β/deg | 109.10(3) |
| γ/deg | 90 |
| V/Å^3^ | 9512(4) |
| Z | 4 |
| *D*_calcd_/g cm^-3^ | 1.181 |
| *μ*/mm^-1^ | 1.004 |
| *F*(000) | 3584 |
| Radiation | Cu *Kα* |
| 2θ range for data collection/deg | 5.34-150.758 |
| Index ranges | -15≤h≤15; -50≤k≤49; -23≤l≤23 |
| Reflections collected | 65144 |
| Independent reflections | 18909 |
| Data/restraints/parameters | 13473/96/1263 |
| Goodness-of-fit on *F*^2^ | 1.025 |
| Final *R* indexes [I>=2*σ* (I)] | 0.0964 |
| Final *R* indexes [all data] | 0.1241 |
| Largest diff. peak/hole/e Å^-3^ | 2.987/-1.152 |


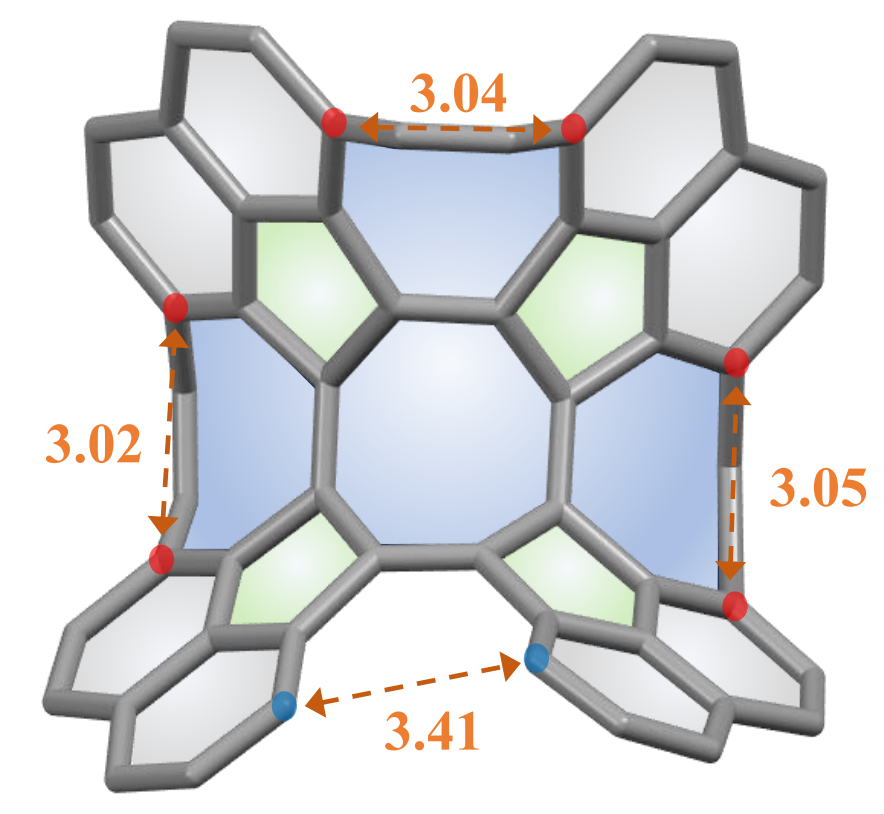


**Figure S21.** Crystal structures of **NH1**. Molecular structure with thermal ellipsoids at 50% probability. Hydrogen atoms, 1;4-*tert*-butylphenyl groups, and solvent molecules are omitted for clarity, Bond lengths.


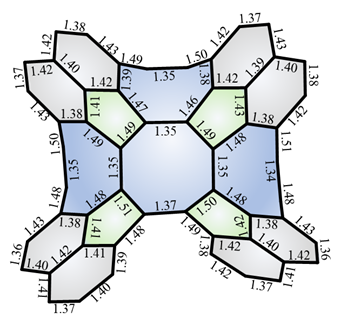


**Figure S22.** Observed bond lengths of **HN1** (mean values, Å). 1; 4-*tert*-butylphenyl groups are omitted for clarity.

The ground- and excited-state geometries of **HN1** were fully optimized with density-functional theory (DFT) and linear-response time-dependent DFT (TDDFT), respectively. Unless otherwise specified, all computational calculations in this study were performed using the B3LYP functional in conjunction with the 6-311G* basis set^3^ in the GAUSSIAN 16 package.^4^  All analyses as well as drawing of various kinds of maps were finished via the Multiwfn code.^5,6^ We probed the nucleusindependent chemical shift (NICS) to explore their aromaticity.^7^


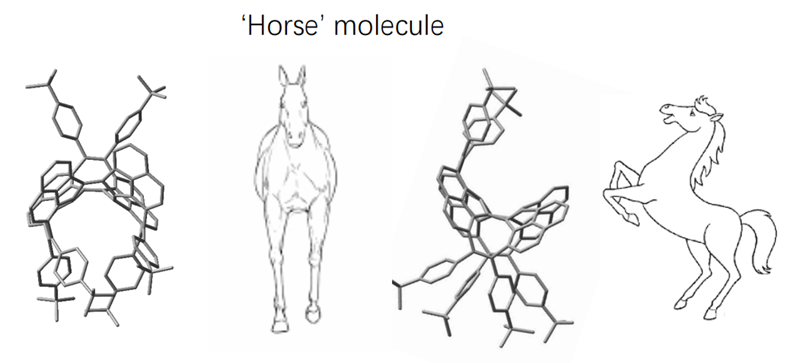


**Figure S23.** Calculated geometry of **HN1**.


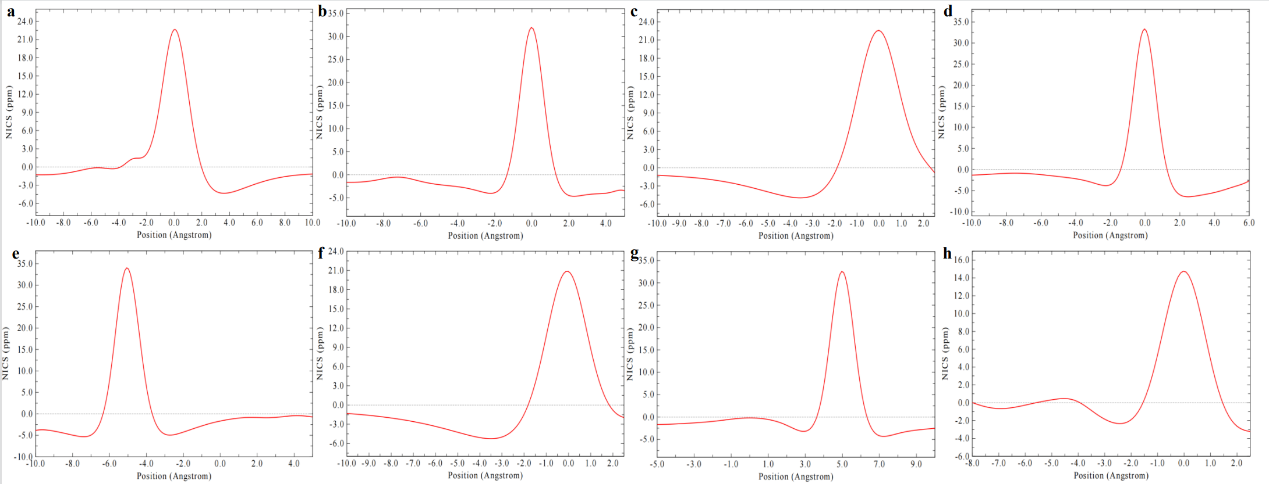


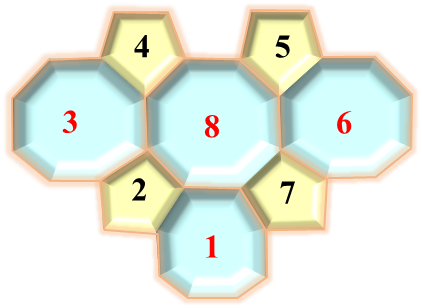


**Figure S24.** Diagram of NICSzz curves of **HN1**. Figures a-h correspond to the NICSzz results for rings 1 to 8 of the **HN1** molecule, respectively. These rings are numbered in a clockwise direction, starting from ring 1, Ring 1 is defined as the ring located directly opposite the position where the outer ring fails to form a complete eight‑membered ring. Moving clockwise in sequence, these rings are successively named Ring 2, Ring 3, Ring 4, Ring 5, Ring 6, Ring 7, and the central octagon is named Ring 8.

**Table S2.** Multicenter bond order for ring 1-8 of **HN1**.

|  | multicenter bond order | normalized multicenter bond order |
| --- | --- | --- |
| Ring 1 | 0.0011406990 | 0.4286929907 |
| Ring 2 | 0.0212662999 | 0.4629545822 |
| Ring 3 | 0.0012241679 | 0.4324940365 |
| Ring 4 | 0.0182763416 | 0.4491359861 |
| Ring 5 | 0.0210302027 | 0.4619220476 |
| Ring 6 | 0.0010463270 | 0.4240903859 |
| Ring 7 | 0.0212595723 | 0.4629252871 |
| Ring 8 | 0.0026611957 | 0.4765786707 |


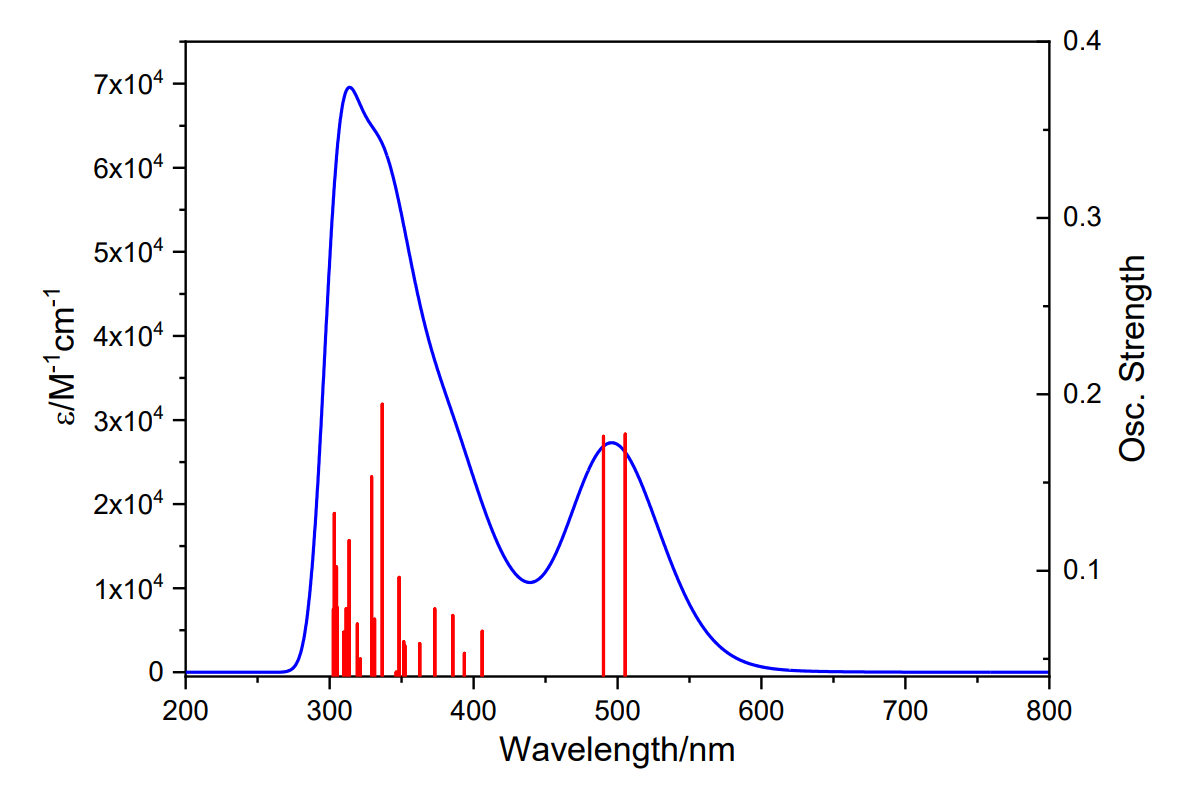


**Figure S25.** Calculated UV-*vis* of **HN1**.

**Figure S26.** Absolute luminescence quantum yield (QY) of **10** and **HN1** in the CH_2_Cl_2_.


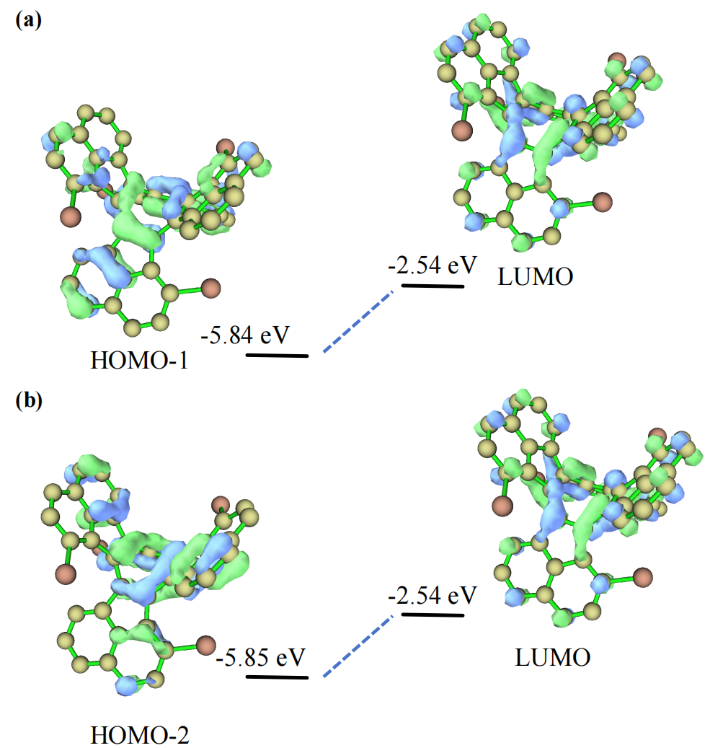


**Figures S27**. HOMOs and LUMOs of **10** related to the observed absorption peakat ca. 451 nm.


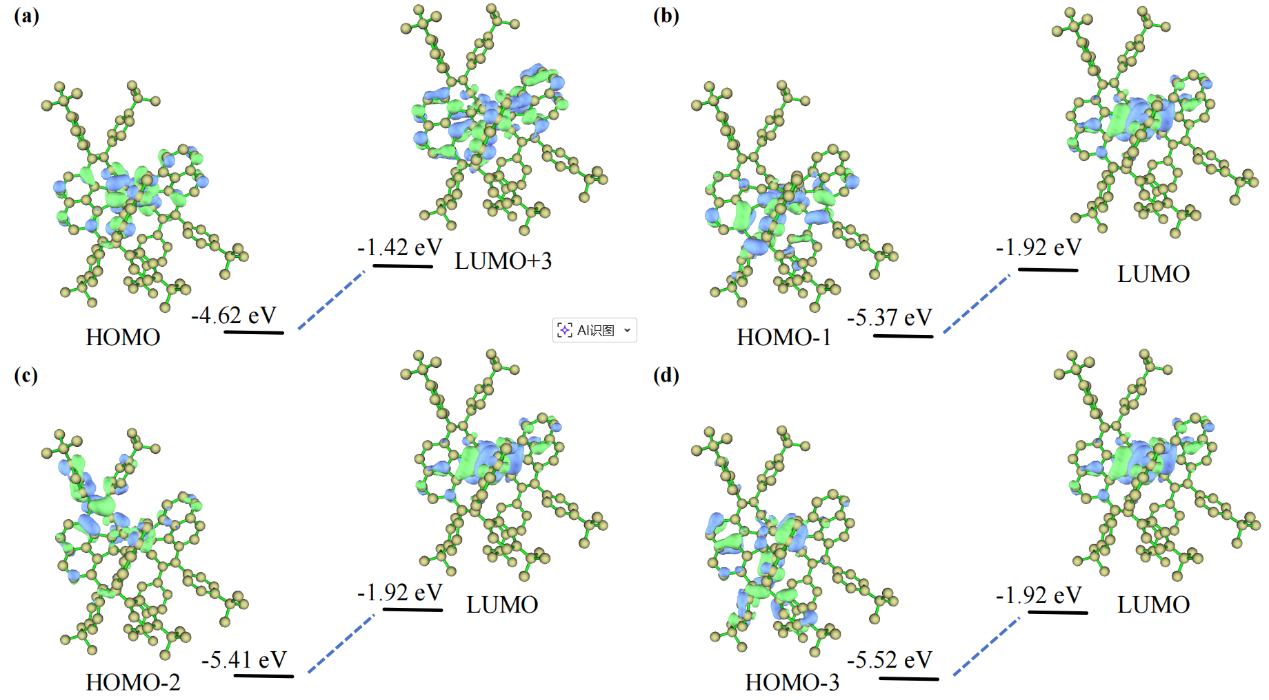


**Figures S28**. HOMOs and LUMOs of **HN1** related to the observed broad shoulder between 400 and 500 nm.

**Table S3.** Selected TD-DFT (B3LYP/6-31G*) calculated wavelength, oscillator strength and compositions of major electronic transitions of **10**.

| Wavelength(nm) | Energy(eV) | Osc. Strength(f) | Majorcontributions |
| --- | --- | --- | --- |
| 590.9 | 2.0982 | 0 | H -> L 99.2% |
| 467.21 | 2.6537 | 0.0371 | H -> L+1 93.6% |
| 467.2 | 2.6538 | 0.0371 | H -> L+2 93.6% |
| 451.71 | 2.7448 | 0.1495 | H-1 -> L 92.3% |
| 451.7 | 2.7448 | 0.1494 | H-2 -> L 92.3% |
| 447.69 | 2.7694 | 0.0012 | H -> L+3 94.2% |
| 397.37 | 3.1201 | 0.0711 | H-3 -> L 56.3%, H-1 -> L+2 13.9%, H-2 -> L+1 13.9%, H-8 -> L 9.6% |
| 396.03 | 3.1307 | 0 | H-1 -> L+1 42.8%, H-2 -> L+2 42.8% |
| 387.27 | 3.2015 | 0.0456 | H-3 -> L 40.3%, H-1 -> L+2 23.5%, H-2 -> L+1 23.5% |
| 384.24 | 3.2268 | 0 | H-2 -> L+1 46.0%, H-1 -> L+2 46.0% |
| 378.48 | 3.2759 | 0.2744 | H-2 -> L+2 46.1%, H-1 -> L+1 46.0% |
| 372.46 | 3.3288 | 0.0602 | H-4 -> L 80.8%, H-1 -> L+3 11.2% |
| 372.45 | 3.3289 | 0.0602 | H-5 -> L 80.8%, H-2 -> L+3 11.2% |
| 368.8 | 3.3618 | 0.0244 | H-1 -> L+3 77.2%, H-4 -> L 12.4% |
| 368.79 | 3.3619 | 0.0244 | H-2 -> L+3 77.2%, H-5 -> L 12.3% |
| 367.68 | 3.372 | 0.0298 | H-8 -> L 82.1%, H-1 -> L+2 6.5%, H-2 -> L+1 6.5% |
| 365 | 3.3968 | 0 | H-6 -> L 90.4% |
| 354.63 | 3.4962 | 0 | H-7 -> L 91.5% |
| 345.15 | 3.5922 | 0.0874 | H-9 -> L 85.7% |
| 345.15 | 3.5922 | 0.0874 | H-10 -> L 85.7% |
| 338.27 | 3.6652 | 0.083 | H-3 -> L+1 90.3% |
| 338.27 | 3.6653 | 0.083 | H-3 -> L+2 90.3% |
| 336.76 | 3.6817 | 0.009 | H-11 -> L 90.6% |
| 328.25 | 3.7771 | 0 | H-4 -> L+1 33.9%, H-5 -> L+2 33.8%, H-3 -> L+3 8.6%, H-4 -> L+2 7.0%, H-5 -> L+1 7.0% |
| 325.56 | 3.8083 | 0.006 | H-5 -> L+1 26.8%, H-4 -> L+2 26.7%, H-5 -> L+2 18.2%, H-4 -> L+1 18.2%, H-11 -> L 5.1% |
| 322.8 | 3.8409 | 0.0062 | H-8 -> L+1 28.0%, H-6 -> L+1 23.6%, H-6 -> L+2 20.4%, H-8 -> L+2 17.1% |
| 322.79 | 3.841 | 0.0062 | H-8 -> L+2 28.0%, H-6 -> L+2 23.6%, H-6 -> L+1 20.5%, H-8 -> L+1 17.1% |
| 321.99 | 3.8505 | 0.0685 | H-4 -> L+1 29.9%, H-5 -> L+2 29.9%, H-4 -> L+2 17.6%, H-5 -> L+1 17.6% |
| 321.23 | 3.8596 | 0 | H-3 -> L+3 79.4%, H-4 -> L+2 6.5%, H-5 -> L+1 6.5% |
| 319.53 | 3.8802 | 0.0469 | H-6 -> L+1 31.5%, H-8 -> L+2 28.4%, H-6 -> L+2 18.9%, H-8 -> L+1 9.4% |
| 319.53 | 3.8803 | 0.0469 | H-6 -> L+2 31.6%, H-8 -> L+1 28.4%, H-6 -> L+1 18.8%, H-8 -> L+2 9.4% |
| 319.07 | 3.8858 | 0 | H-5 -> L+1 31.5%, H-4 -> L+2 31.5%, H-5 -> L+2 11.5%, H-4 -> L+1 11.4% |
| 311.25 | 3.9835 | 0.1008 | H-7 -> L+1 65.2%, H-5 -> L+3 12.4%, H-4 -> L+3 12.0% |
| 311.24 | 3.9835 | 0.1008 | H-7 -> L+2 65.2%, H-4 -> L+3 12.4%, H-5 -> L+3 12.0% |
| 308.95 | 4.013 | 0.0079 | H-4 -> L+3 67.9%, H-7 -> L+2 10.3%, H-7 -> L+1 9.9% |
| 308.95 | 4.0131 | 0.008 | H-5 -> L+3 68.0%, H-7 -> L+1 10.2%, H-7 -> L+2 9.9% |
| 308.88 | 4.014 | 0 | H-8 -> L+3 39.0%, H-10 -> L+1 12.9%, H-9 -> L+2 12.8%, H-9 -> L+1 9.4%, H-10 -> L+2 9.4% |
| 308.3 | 4.0215 | 0.0243 | H-6 -> L+3 60.2%, H-9 -> L+2 10.5%, H-10 -> L+1 10.5% |
| 307.18 | 4.0362 | 0.0399 | H-6 -> L+3 31.1%, H-9 -> L+1 17.5%, H-10 -> L+2 17.4%, H-7 -> L+3 13.9%, H-10 -> L+1 5.4%, H-9 -> L+2 5.4% |
| 303.75 | 4.0818 | 0 | H-10 -> L+1 30.4%, H-9 -> L+2 30.3%, H-8 -> L+3 16.6%, H-10 -> L+2 7.2%, H-9 -> L+1 7.2% |
| 302.43 | 4.0996 | 0.0012 | H-9 -> L+2 28.0%, H-10 -> L+1 28.0%, H-9 -> L+1 18.5%, H-10 -> L+2 18.3%, H-6 -> L+3 5.8% |
| 301.75 | 4.1088 | 0 | H-8 -> L+3 32.2%, H-10 -> L+2 31.7%, H-9 -> L+1 31.6% |
| 298.22 | 4.1575 | 0.0744 | H-11 -> L+1 81.8%, H-10 -> L+3 7.9% |
| 298.22 | 4.1575 | 0.0744 | H-11 -> L+2 81.8%, H-9 -> L+3 7.9% |
| 296.51 | 4.1815 | 0.0015 | H-7 -> L+3 76.8%, H-10 -> L+2 7.3%, H-9 -> L+1 7.3% |
| 290.99 | 4.2608 | 0.005 | H-9 -> L+3 56.5%, H-10 -> L+3 26.7%, H-11 -> L+1 5.6%, H-11 -> L+2 5.3% |
| 290.99 | 4.2608 | 0.005 | H-10 -> L+3 56.5%, H-9 -> L+3 26.7%, H-11 -> L+2 5.6%, H-11 -> L+1 5.3% |
| 289.6 | 4.2811 | 0.0379 | H -> L+4 95.7% |
| 289.6 | 4.2812 | 0.0379 | H -> L+5 95.7% |
| 287.48 | 4.3128 | 0.064 | H -> L+6 95.4% |

**Table S4**: Selected TD-DFT (B3LYP/6-31G*) calculated wavelength, oscillator strength and compositions of major electronic transitions of **HN1**.

| Wavelength(nm) | Energy(eV) | Osc. Strength(*f*) | Major contributions |
| --- | --- | --- | --- |
| 609.11 | 2.0355 | 0.0011 | H→L 99.1% |
| 505.39 | 2.4532 | 0.1775 | H→L+1 94.1% |
| 490.26 | 2.529 | 0.1762 | H→L+2 92.0% |
| 459.6 | 2.6977 | 0.0254 | H→L+3 93.5% |
| 433.34 | 2.8611 | 0.0284 | H-1→L 95.4% |
| 423.4 | 2.9283 | 0.0389 | H-2→L 90.3% |
| 405.93 | 3.0543 | 0.0657 | H-3→L 85.5% |
| 393.65 | 3.1496 | 0.0532 | H-4→L 58.2%,H-1→L+2 14.6%,  H-6→L 8.8%,H-2→L+2 8.2% |
| 393.08 | 3.1542 | 0.0307 | H-1→L+1 63.9%,H-2→L+1 9.5%,  H-6→L 8.4%,H-3→L+1 6.1% |
| 385.59 | 3.2154 | 0.0747 | H-2→L+1 67.1%,H-1→L+1 6.8% |
| 382.12 | 3.2447 | 0.0141 | H-1→L+2 50.5%,H-4→L 22.3%,  H-2→L+2 12.1%,H-1→L+1 8.3% |
| 379.14 | 3.2701 | 0.0252 | H-3→L+1 34.4%,H→L+4 33.6%,  H-5→L 6.5% |
| 374.13 | 3.3139 | 0.0068 | H-6→L 24.8%,H-3→L+1 23.0%,  H-2→L+2 19.3%,H-5→L 9.5%,  H→L+4 6.8% |
| 373.63 | 3.3183 | 0.021 | H-2→L+2 43.1%,H→L+4 18.6%,  H-1→L+2 7.7%,H-3→L+1 6.0%,  H-6→L 5.6% |
| 373.09 | 3.3232 | 0.0783 | H-5→L 28.4%,H→L+4 28.3%,  H-6→L 13.7%,H-3→L+1 8.3%,  H-1→L+1 6.0% |
| 367.67 | 3.3722 | 0.033 | H-5→L 45.2%,H-6→L 31.5%,  H-3→L+1 6.6%,H-2→L+1 5.7% |
| 364.65 | 3.4 | 0.0047 | H-3→L+2 47.2%,H-1→L+3 22.3%,  H-4→L+1 5.8%,H→L+5 5.8% |
| 362.57 | 3.4196 | 0.0588 | H→L+5 77.9%,H-1→L+3 9.9% |
| 357.72 | 3.4659 | 0.037 | H-3→L+2 37.2%,H-1→L+3 32.0%,  H→L+6 8.0%,H-4→L+1 6.1% |
| 355.26 | 3.49 | 0.0197 | H-4→L+1 27.9%,H-2→L+3 22.9%,  H-1→L+3 17.3%,H-3→L+1 7.2% |
| 352.29 | 3.5194 | 0.0575 | H→L+6 36.6%,H-2→L+3 18.7%,  H→L+7 11.1%,H-4→L+2 8.5%,  H-5→L+1 6.2% |
| 351.53 | 3.527 | 0.0598 | H-4→L+1 43.7%,H-2→L+3 29.5%,  H→L+6 7.4%,H-3→L+3 5.4% |
| 348.3 | 3.5597 | 0.0963 | H-4→L+2 50.5%,H→L+7 29.8%,  H-5→L+1 5.6% |
| 346.23 | 3.581 | 0.0425 | H→L+7 28.8%,H-3→L+3 21.9%,  H→L+6 16.7%,H-4→L+2 16.1% |
| 344.97 | 3.5941 | 0.0302 | H-3→L+3 24.1%,H-5→L+2 22.4%,  H→L+6 15.0%,H-5→L+1 14.9% |
| 342.23 | 3.6229 | 0.002 | H-7→L 78.9%,H-5→L+1 7.4% |
| 340.2 | 3.6444 | 0.0268 | H-5→L+1 52.0%,H→L+7 14.0%,  H-7→L 13.3%,H-5→L+2 7.5% |
| 339.15 | 3.6557 | 0.01 | H-5→L+2 31.0%,H-3→L+3 25.6%,  H-4→L+2 9.1%,H→L+8 8.0%,  H-2→L+3 7.2% |
| 336.58 | 3.6836 | 0.1945 | H-6→L+1 90.5% |
| 331.97 | 3.7348 | 0.0145 | H-8→L 78.7%,H-6→L+2 6.6% |
| 331.28 | 3.7426 | 0.0726 | H-6→L+2 41.4%,H→L+8 31.2%,  H-8→L 5.5% |
| 329.17 | 3.7666 | 0.1533 | H→L+8 38.0%,H-6→L+2 31.5%,  H-5→L+2 12.1% |
| 326.87 | 3.7931 | 0.0072 | H-9→L 73.1%,H-4→L+3 10.3% |
| 325.31 | 3.8112 | 0.0172 | H-4→L+3 63.8%,H-9→L 11.5%,  H-6→L+2 6.9% |
| 321.29 | 3.859 | 0.05 | H-5→L+3 37.5%,  H-7→L+1 23.1%,H-4→L+3 6.2% |
| 319.19 | 3.8843 | 0.0698 | H-7→L+1 35.7%,  H-5→L+3 32.0%,H-7→L+2 5.3% |
| 315.18 | 3.9338 | 0.0332 | H-10→L 72.3%,H-11→L 11.6% |
| 313.62 | 3.9534 | 0.1171 | H-11→L 45.1%,H-10→L 7.9%,  H-16→L 7.9%,H-12→L 6.0%,  H-13→L 5.8% |
| 311.45 | 3.9809 | 0.0786 | H-8→L+2 13.6%,  H-5→L+3 12.4%,H-13→L 9.9%,  H-12→L 9.5%,H-6→L+3 9.3%,  H-7→L+2 6.2%,H→L+1 05.6%,  H-15→L 5.2% |
| 310.97 | 3.9871 | 0.0009 | H-6→L+3 66.0%,H-7→L+2 7.7% |
| 309.77 | 4.0024 | 0.0652 | H-12→L 21.3%,H→L+10 14.3%,  H-8→L+2 8.8%,H-1→L+4 5.8%,  H-15→L 5.4% |
| 308.88 | 4.014 | 0.0326 | H-7→L+2 53.3%,H-6→L+3 7.5%,  H-8→L+1 7.3%,H-13→L 6.2%,  H-7→L+1 5.1% |
| 308.5 | 4.019 | 0.007 | H-8→L+1 25.9%,H→L+9 16.4%,  H-7→L+2 15.1%,H-1→L+4 10.8% |
| 308.02 | 4.0251 | 0.0193 | H-13→L 27.0%,H-11→L 20.0%,  H-12→L 8.6% |
| 305.65 | 4.0564 | 0.0171 | H-12→L 24.7%,H-15→L 20.6%,  H-16→L 7.3%,H→L+9 6.7% |
| 305.06 | 4.0643 | 0.0794 | H-14→L 24.1%,H-2→L+4 9.9%,  H-12→L 9.3%,H→L+9 8.6%,  H-8→L+1 6.5%,H-13→L 5.2% |
| 304.81 | 4.0676 | 0.1022 | H-8→L+1 22.4%,  H-1→L+4 22.2%,H-1→L+5 9.7%,  H-2→L+4 8.5%,H-12→L 5.1% |
| 303.26 | 4.0884 | 0.1325 | H-15→L 22.3%,  H-1→L+4 14.8%,H-14→L 11.1%,  H→L+9 10.5%,H-13→L 8.4% |
| 302.61 | 4.0971 | 0.0781 | H-2→L+4 17.9%,H-14→L 15.5%,  H-9→L+2 11.1%,H→L+9 8.4%,  H-8→L+2 6.0%,H-9→L+1 5.5% |
| 301.69 | 4.1097 | 0.0055 | H-9→L+1 44.3%,  H-17→L 11.8%,H-14→L 8.0% |

**5. Optoelectronic Devices**

Experimental Section

Materials

ITO substrates were obtained from PsiOTech Ltd. Phenyl-C61-butyric acid methyl ester (PCBM) (>99.5%) was bought from Luminescence Technology Crop. NiOx and Me4-PACz were purchased from Liaoning Youxuan Crop and TCI, respectively. Lead (II) iodide (>98%), Cesium chloride (99.999%, metals basis), and Bathocuproine (BCP) were obtained from TCI company. Formamidinium iodide (FAI) was purchased from Greatcellsolar Materials. Anhydrous Chlorobenzene, Dimethylformamide (DMF), N-methyl-2-pyrrolidone (NMP), and Isopropanol (IPA) were bought from Acros Organics. All materials were used without more purification.

Precursor preparation

For NiOx precursors, it was prepared as reported before.^8^ 10 mg NiOx powder was dispersed in the 1 mL DI water and ultrasonicated at 40 °C for 10 min, then filtered with a 0.25 um hydrophilic filter. For Me-4PACz precursors, 1.5 mg Me-4PACz powder was dissolved in the 1 mL isopropanol and then ultrasonicated at 40 °C for 10 min. For perovskite precursors, the 1.6 M presynthesized Cs_0.15_FA_0.85_PbI_0.85_Cl_0.15_ were dissolved in DMF:NMP (960:150) mixed solvent and then put on the hotplate at 70 °C overnight. For the perovskite with additives, different amounts of **10** and **HN1** (0.25 mg, 0.5 mg, 1.0 mg, and 2.0 mg) were dissolved in 1 mL DMF and NMP mixed solvents. 20 mg PCBM powder was dissolved in 1 mL chlorobenzene solvent and 0.5 mg BCP powder was dissolved in 1 mL isopropanol. Both of them were put on a hotplate at 70 °C for stirring overnight.

Device fabrication

Patterned ITO was rinsed with acetone to remove the protective glue. Then they were ultrasonically cleaned with 2% Hellmanex detergent, deionized water, acetone, and isopropanol for 20 mins, respectively, followed dry with a nitrogen gun. For Nickel oxide, the clean ITO substrates were plasma treated for 15 min and then coated with NiOx aqueous at 5000 rpm for the 40 s and annealed in ambient air at 120 °C for 10 min. Then, the Me-4PACz solutions were deposited on as-prepared NiOx film at 4000 rpm for 30 s and annealed in glovebox at 100 °C for 10 min. Subsequently, substrates were transferred to the home-made dry box. For perovskite active layer, 25 μL precursors were spin-coated on the substrates at 5000 rpm for 40 s, followed by gas blowing for 30 s, annealing at 60 °C for 2 min, then 150 °C for 10 min. Subsequently, the as-prepared perovskite films were transferred to a nitrogen-filled glovebox for electron transport layer deposition. 25 μL PCBM solutions were dynamically spin-coated at 2000 rpm for 30 s, followed by annealing at 100 °C for 3 min. Finally, 35 μL BCP was dynamically deposited on substrates at 4000 rpm for 30 s, followed by an 80 nm thermally evaporated Ag cathode (Mantis evaporator, base pressure of 10^-7^ mbar).

Photovoltaic Device Characterization

The devices’ EQE spectra were acquired by utilizing the monochromatic light emitted by a halogen lamp ranging from 350 nm to 800 nm. The NIST-traceable Si diode (Thorlabs) was used to calibrate the reference spectra. The *J-V* characteristics were obtained using a computer-controlled Keithley 2450 source measure unit at a solar simulator of the Abet Sun 3000 Class AAA model. The intensity of the incident light was calibrated using a Si reference cell (NIST traceable, VLSI) and adjusted by measuring the spectral mismatch factor between a real solar spectrum, the spectral response of the reference cell, and perovskite devices. All devices were scanned from short circuit to forward bias (1.3 V) to and reverse with a rate of 0.025 V s^−1^. No treatment was applied before measurements. The active area for all devices was 4.5 mm^2^.

**Table S5**. Photovoltaic performance parameters of champion perovskite devices with different additives.

| **Device** | **Scan direction** | **Voc [V]** | **Jsc [mA/cm^2^]** | **FF [%]** | **PCE [%]** |
| --- | --- | --- | --- | --- | --- |
| **Ref** | Forward | 1.10 | 24.15 | 81.33 | 21.65 |
|  | Reverse | 1.10 | 24.15 | 77.68 | 20.60 |
| **10** | Forward | 1.13 | 23.65 | 83.11 | 22.15 |
|  | Reverse | 1.13 | 23.65 | 80.70 | 21.65 |
| **HN1** | Forward | 1.13 | 24.32 | 82.28 | 22.61 |
|  | Reverse | 1.14 | 24.32 | 81.08 | 22.39 |

**Table S6.** Photovoltaic parameters of perovskite devices with additives from literature.^9-15^

| Device | *V*oc[V] | *J*sc[mA·cm^2^] | FF[%] | PCE[%] |
| --- | --- | --- | --- | --- |
| PNDI-FBT | 0.88 | 22.49 | 78.55 | 15.57 |
| FREA(INIC) | 1.14 | 24.58 | 77.55 | 21.70 |
| FGQDs | 1.15 | 24.53 | 77.07 | 21.74 |
| **HN1** | **1.13** | **24.32** | **82.28** | **22.61** |
| PVK-BG-0.5 | 1.19 | 24.47 | 77.72 | 22.72 |
| Dec-oxoG | 1.16 | 24.20 | 84.10 | 23.70 |
| o-TB-GDY | 1.20 | 25.82 | 82.88 | 25.62 |
| JJ24 co-SAM | 1.18 | 26.18 | 86.75 | 26.98 |


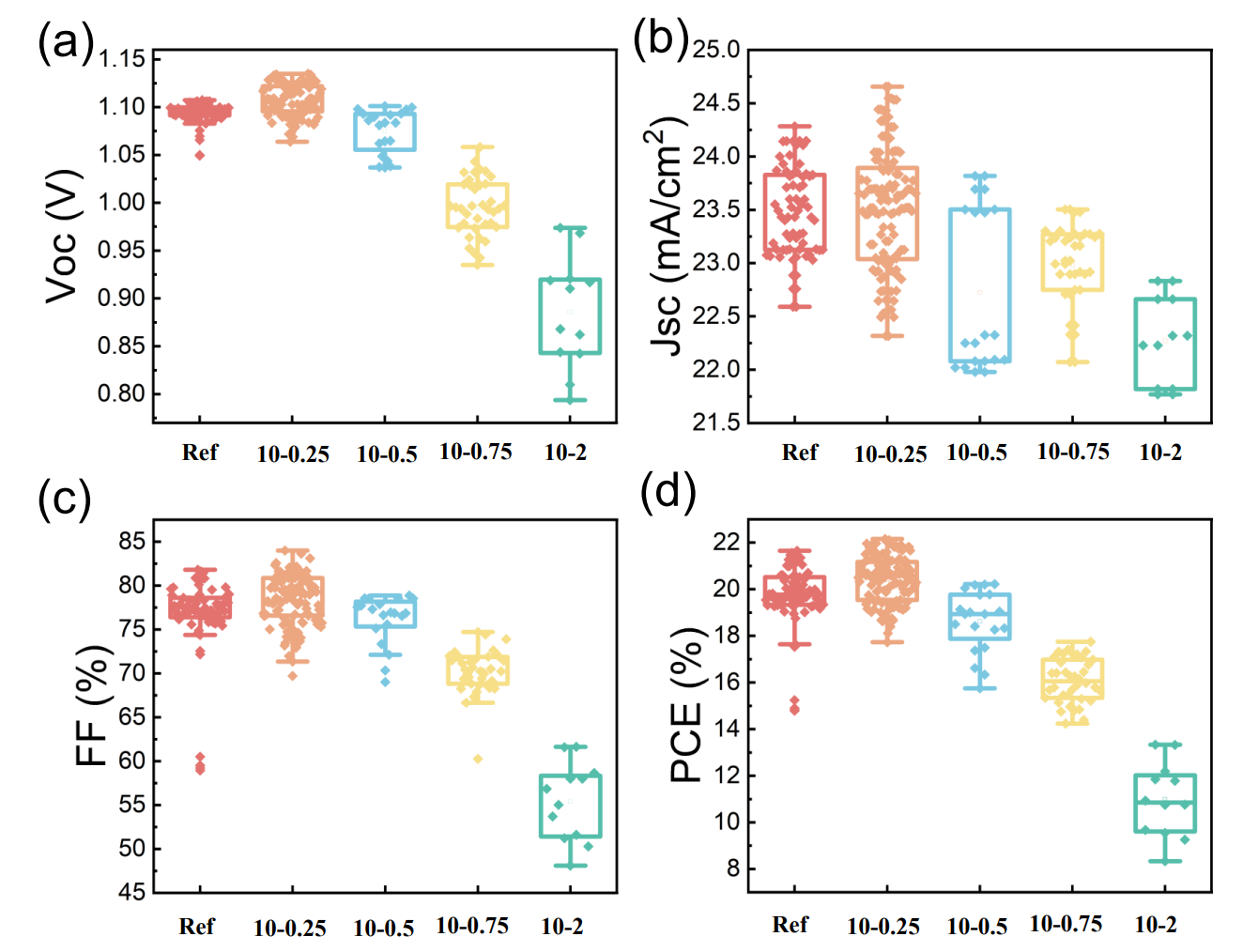


**Figure S29.** Photovoltaic performance parameters: (a) V_OC_, (b) J_SC_, (c) FF, and (d) PCE distributions of perovskite devices with **10** additives. A total of 268 devices were measured.


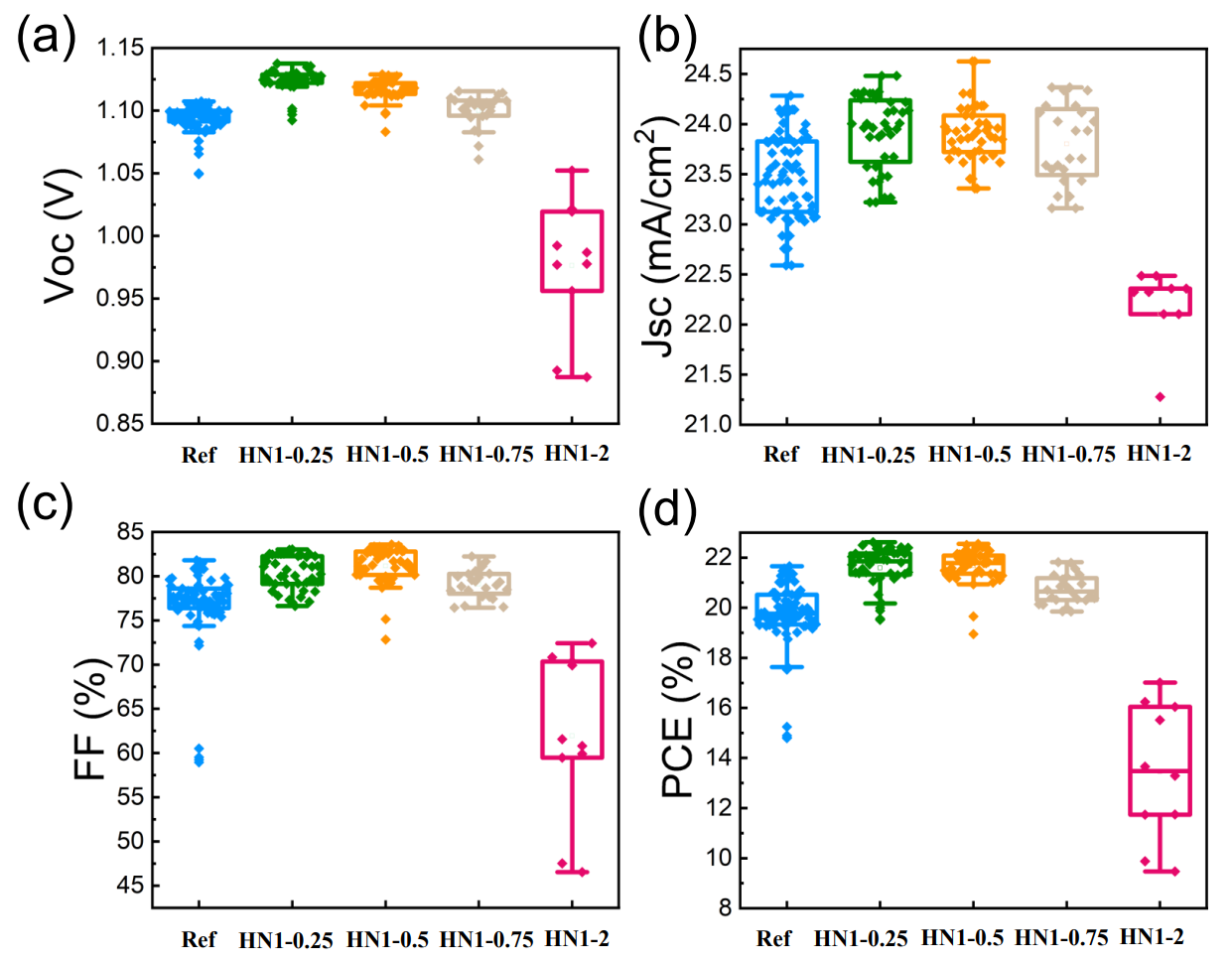


**Figure S30.** Photovoltaic performance parameters: (a) V_OC_, (b) J_SC_, (c) FF, and (d) PCE distributions of perovskite devices with **HN1** additives. A total of 188 devices were measured.


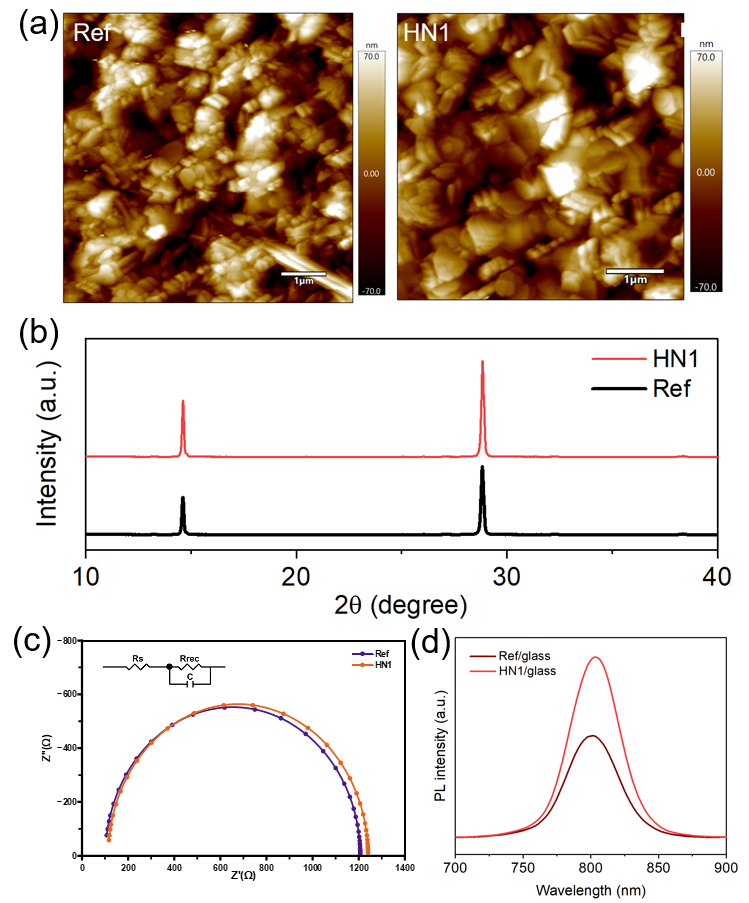


**Figure S31.** (a) AFM topography images of perovskite layers grown on the Ref and HN1-modified substrates. (b) XRD diffraction patterns of perovskite films grown on the Ref and **HN1**-modified substrates. (c) Nyquist plots (inset: equivalent circuit) of the Ref and **HN1**-modified samples measured in the dark. (d) PL spectra of perovskite films grown on the Ref and **HN1**-modified substrates.


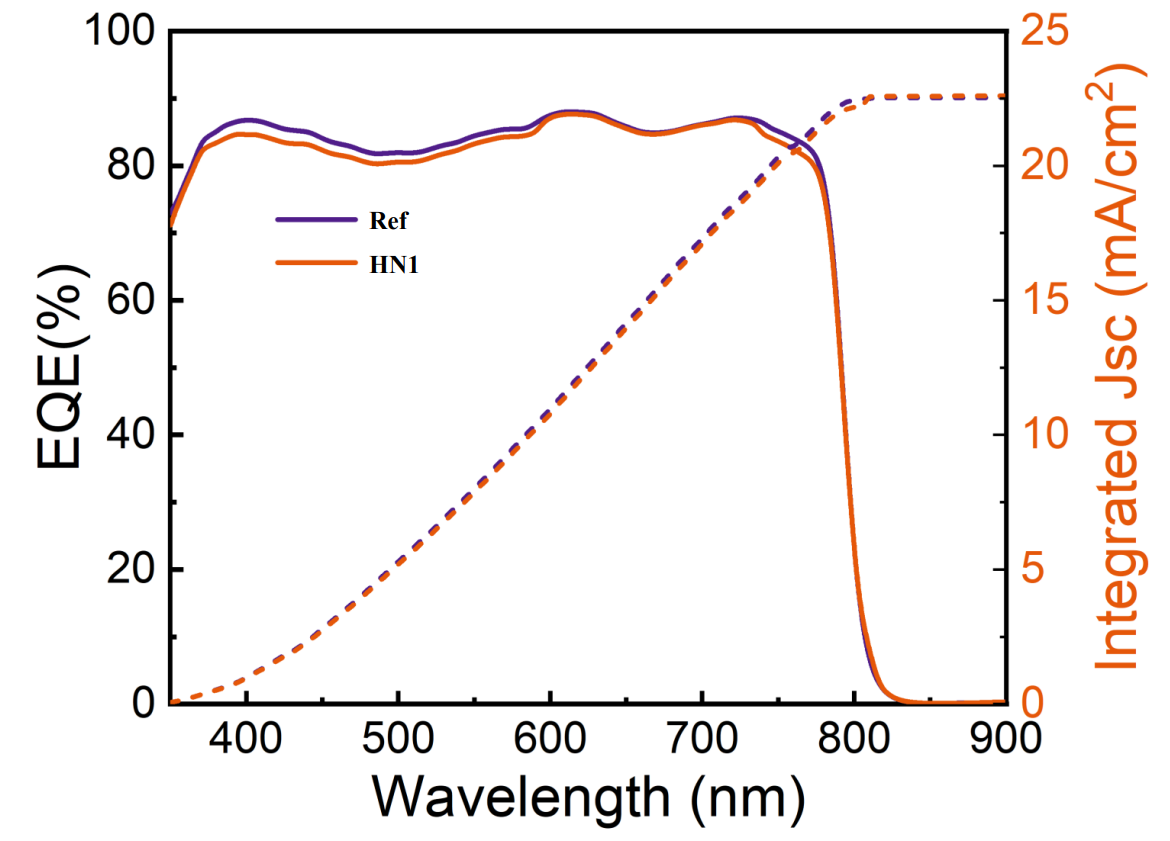


**Figure S32.** EQE curves of devices with or without **HN1** additives.


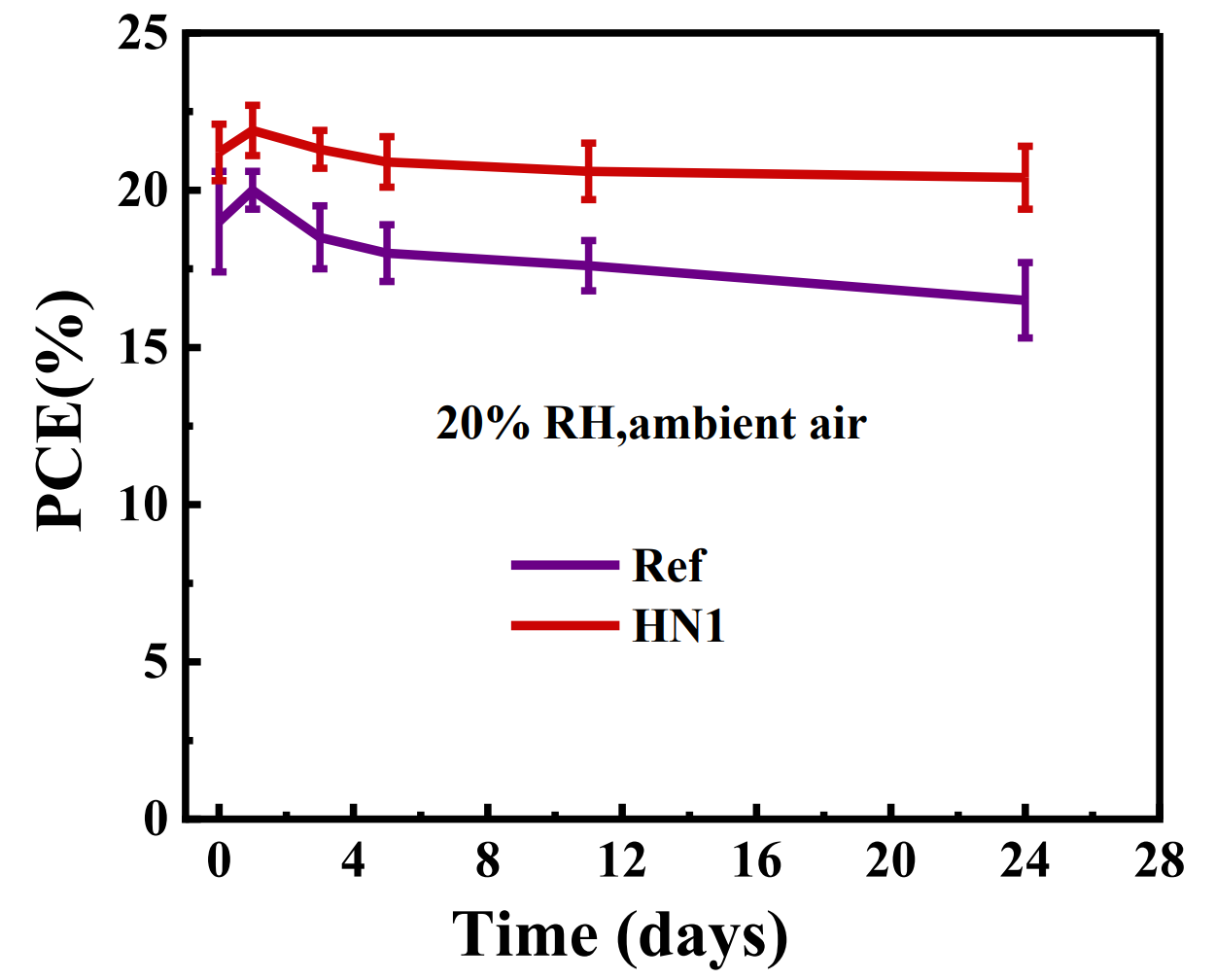


**Figure S33.** Evolution of the PCE of unencapsulated perovskite devices stored under ambient conditions (25 °C, 20% RH, dim light).

1. **Reference:**
2. Shaik, F. H.; Kar, G. K. Beilstein. Studies on polynuclear furoquinones. Part 1: Synthesis of tri- and tetra-cyclic furoquinones simulating BCD/ABCD ring system of furoquinone diterpenoids. *J. Org. Chem.,* **2009**, 5, No. 47.
3. a) K. Y. Cheung, C. K. Chan, Z. Liu, Q. Miao, *Angew. Chem. Int. Ed.* **2017**, *56*, 9003-9007; b) Y. Duan, M. Chen, H. Hayashi, H. Yamada, X. Liu, L. Zhang, *Chem. Sci.* **2023**, *14*, 10420-10428; c) R. Fu, X. Chen, F. Qiu, X. Liu, J. Xia, L. Zhang, *Angew. Chem. Int. Ed.* **2025**, *64*, e202420419; d) A. Konishi, H. Tokuyama, H. Kitamura, S. Tsuchida, M. Suzuki, K.-i. Nakayama, R. Kishi, M. Yasuda, *J. Am. Chem. Soc.* **2025**, *147*, 17281-17292; e) S. Matsubara, Y. Koga, Y. Segawa, K. Murakami, K. Itami, *Nat. Catal.* **2020**, *3*, 710-718; f) R. W. Miller, S. E. Averill, S. J. Van Wyck, A. C. Whalley, *J. Org. Chem.* **2016**, *81*, 12001-12005; g) S. Nobusue, Y. Tobe, *Synlett* **2016**, *27*, 2140-2144; h) M.-W. Wang, J. Chan, Q. Long, Y. Wagenhäuser, F. Würthner, W. Jiang, Z. Wang, *J. Am. Chem. Soc.* **2025**, *147*, 47102-47108.
4. a) McLean, A. D.; Chandler, G. S. Contracted Gaussian basis sets for molecular calculations. I. Second row atoms, Z=11-8, *J. Chem. Phys*., **1980**, 72, 5639-5648; (b) K. Raghavachari, J. S. Binkley, R. Seeger, and J. A. Pople, Self‐consistent molecular orbital methods. A basis set for correlated wave functions. *J. Chem. Phys.,* **1980**, 72, 650-654.
5. M. J. Frisch, G. W. Trucks, H. B. Schlegel, G. E. Scuseria, M. A. Robb, J. R. Cheeseman, G. Scalmani, V. Barone, G. A. Petersson, H. Nakatsuji, X. Li, M. Caricato, A. V. Marenich, J. Bloino, B. G. Janesko, R. Gomperts, B. Mennucci, H. P. Hratchian, J. V. Ortiz, A. F. Izmaylov et al., Gaussian 16 Rev. C.01 (Wallingford, CT, 2016).
6. Tian Lu, Feiwu Chen, Multiwfn: A Multifunctional Wavefunction Analyzer, *J. Comput. Chem.,* **2012,** 33, 580-592.
7. Tian Lu, A comprehensive electron wavefunction analysis toolbox for chemists, Multiwfn, *J. Chem. Phys.,* **2024,** 161, 082503.
8. Schleyer, P. v. R.; Maerker, C.; Dransfeld, A.; Jiao, H.; van Eikema Hommes, N. J. Nucleus-Independent Chemical Shifts: A Simple and Efficient Aromaticity Probe. *J. Am. Chem. Soc.,* **1996,** 118, 6317-6318.
9. Zhang, Z; Ran Ji, R; J. Hofstetter, Y.; Deconinck, M; Brunner, J; Li, Y; An, Q; Vaynzof, Y. Towards Low-Temperature Processing of Efficient γ-CsPbI 3 Perovskite Solar Cells. *J. Mater. Chem. A,* **2023**, 11, 16115-16126.
10. Shen, T.; Li, T.; Wang, Y.; Yuan, C.; Zhao, Y.-H.; Yan, D.; Liang, J.; Liu, Y.; Jen, A. K.-Y. Boosting Tin-Perovskite Solar Cell Efficiency Using Direct Arylation Polymerized *n*-Type Organic Semiconductors as Superior Electron-Transporting Layers. *ACS Energy Lett.* **2025**.
11. Jiang, Y.; Zai, H.; Zheng, X.; Zhang, Y.; Faheem, M. B.; Dai, S.; Lv, Z.; Ma, Y.; Li, L.; Tao, Y.; Zhu, X.; Kang, J.; Liu, S.; Lu, H.; Qin, S.; Song, T.; Wang, X.; Xiao, X.; Yang, H.; Lu, X.; Qiao, Q.; Chen, Q.; Zhou, H.; Zhan, X. Fused-Ring Electron Acceptors as a Versatile Additive Platform for Efficient Perovskite Photovoltaics. *J. Am. Chem. Soc.* **2025**.
12. Chen, Y.; Chen, J.; Wang, X.; Deng, P.; Shen, Y.; Wang, X. Fluorinated Graphene Quantum Dots-Induced Defect Passivation of Perovskite Film toward Stable and Efficient Perovskite Solar Cell. *Int. J. Hydrogen Energy* **2025**.
13. Li, G.; Hu, Y.; Li, M.; Tang, Y.; Zhang, Z.; Musienko, A.; Cao, Q.; Akhundova, F.; Li, J.; Prashanthan, K.; Yang, F.; Janasik, P.; Appiah, A. N. S.; Trofimov, S.; Livakas, N.; Zuo, S.; Wu, L.; Wang, L.; Yang, Y.; Agyei-Tuffour, B.; MacQueen, R. W.; Naydenov, B.; Unold, T.; Unger, E.; Aktas, E.; Eigler, S.; Abate, A. Managing Excess Lead Oxide with Functionalized Oxo-Graphene Nanosheets for Stable Perovskite Solar Cells. *Angew. Chem., Int. Ed.* **2023**, 135, e202307395.
14. Fan, L.; Wang, L.; Bi, C.; Zhao, W.; Li, M.; Li, S.; Wang, F.; Cao, J.; Liu, X.; Wu, Q.; Liu, H.; Yang, L. Customizing a Chemical and Field-Effect Passivation Strategy for Efficient and Durable Perovskite Solar Cells Using Inorganic 2D/2D Nanocomposites. *Small*, **2025**, 22, e12984.
15. Shao, C.; He, J.; Ma, J.; Wang, Y.; Niu, G.; Zhang, P.; Yang, K.; Zhao, Y.; Wang, F.; Li, Y.; Wang, J. Multifunctional Graphdiyne Enables Efficient Perovskite Solar Cells via Anti Solvent Additive Engineering. *Nano-Micro Lett.* **2025**, 17, 121.
16. Jiang, W.; Qu, G.; Huang, X.; Chen, X.; Chi, L.; Wang, T.; Wong, C.-T.; Lin, F. R.; Yang, C.; Jiang, Q.; Wu, S.; Zhang, J.; Jen, A. K.-Y. Toughened Self-Assembled Monolayers for Durable Perovskite Solar Cells. *Nature*, **2025**, 646, 95-101.
